# The Kunitz Domain I of Hepatocyte Growth Factor Activator Inhibitor-2 Inhibits Matriptase Activity and Invasive Ability of Human Prostate Cancer Cells

**DOI:** 10.1038/s41598-017-15415-4

**Published:** 2017-11-08

**Authors:** Shang-Ru Wu, Chen-Hsin Teng, Ya-Ting Tu, Chun-Jung Ko, Tai-Shan Cheng, Shao-Wei Lan, Hsin-Ying Lin, Hsin-Hsien Lin, Hsin-Fang Tu, Pei-Wen Hsiao, Hsiang-Po Huang, Chung-Hsin Chen, Ming-Shyue Lee

**Affiliations:** 1 Department of Biochemistry and Molecular Biology, College of Medicine, National Taiwan University, Taipei, Taiwan; 2 Agricultural Biotechnology Research Center, Academia Sinica, Taipei, Taiwan; 3 Graduate Institute of Medical Genomics and Proteomics, College of Medicine, National Taiwan University, Taipei, Taiwan; 4 Department of Urology, National Taiwan University Hospital, Taipei, Taiwan

## Abstract

Dysregulation of pericellular proteolysis is often required for tumor invasion and cancer progression. It has been shown that down-regulation of hepatocyte growth factor activator inhibitor-2 (HAI-2) results in activation of matriptase (a membrane-anchored serine protease), human prostate cancer cell motility and tumor growth. In this study, we further characterized if HAI-2 was a cognate inhibitor for matriptase and identified which Kunitz domain of HAI-2 was required for inhibiting matriptase and human prostate cancer cell motility. Our results show that HAI-2 overexpression suppressed matriptase-induced prostate cancer cell motility. We demonstrate that HAI-2 interacts with matriptase on cell surface and inhibits matriptase proteolytic activity. Moreover, cellular HAI-2 harnesses its Kunitz domain 1 (KD1) to inhibit matriptase activation and prostate cancer cell motility although recombinant KD1 and KD2 of HAI-2 both show an inhibitory activity and interaction with matriptase protease domain. The results together indicate that HAI-2 is a cognate inhibitor of matriptase, and KD1 of HAI-2 plays a major role in the inhibition of cellular matritptase activation as well as human prostate cancer invasion.

## Introduction

Metastasis is a leading cause of cancer-related mortality. During metastasis, cancer cells often acquire an invasive ability to penetrate through tissue compartments, intravasation, extravasation and growth in a distant region^1–3^. Several pericellular proteases involved in cancer cell motility, tumor invasive growth, and metastasis have been shown to be dysregulated^1–3^. One of the key reasons for the dysregulation of pericellular proteolysis is the imbalance between proteases and their cognate protease inhibitors, which leads to the progression of cancer metastasis^4–7^.

Among pericellular proteases, human matriptase (also named as MT-SP1, TADG-15, ST14) receives much attention because of its role in tumorigenesis and cancer progression^8,9^. Matriptase is a type II transmembrane serine protease that was first identified in breast cancer and is named according to its capability of matrix degradation^10,11^. Matriptase is found in many types of epithelial cells^12,13^ and is required for the epidermal development as well as homeostasis of immune system^14^. Matriptase also participates in the connection of coagulation cascade to epithelial signaling upon tissue repairing^15^. Since several substrates of matriptase, such as urokinase (uPA), stromelysin (MMP3), pro-HGF (hepatocyte growth factor), and PAR2 (protease-activated receptor 2), were implicated in cancer progression^16–18^, the role of matriptase in cancers has been intensely addressed recently. Indeed, a growing number of clinical reports have indicated the involvement of matriptase in various cancer progressions, such as esophageal squamous cell carcinoma, as well as ovarian, cervical, breast, and prostate cancer^19–30^. In addition, the inhibition of matriptase through the use of curcumin has been shown to suppress prostate tumor growth and metastasis^31^. These findings reveal that matriptase exhibits an oncogenic potential and may serve as a target in cancer therapy.

Matriptase has a cognate inhibitor named HAI-1 (Hepatocyte growth factor activator inhibitor 1). HAI-1 is primarily identified as an inhibitor of HGFA (Hepatocyte growth factor activator)^32,33^ and plays an important role in placental development^34–36^. Matriptase and HAI-1 are co-expressed in many epithelial cells^37^, and the regulation of matriptase by HAI-1 is required for epidermal integrity^38^. HAI-1 is a transmembrane bi-Kunitz-type serine protease inhibitor that contains two Kunitz domains (KD1 and KD2)^32^. Further studies manifest that the functional characterization and crystal structure of HAI-1 represses and interacts with matriptase through its KD1^39,40^. Although HAI-1 features as an inhibitor repressing matriptase, it also has a special function in facilitating the trafficking, maturation and activation of matriptase^41,42^. The matriptase-HAI-1 complex is thus regarded as an activated form of cellular matriptase^43,44^. Matriptase and HAI-1 have also been detected in several types of tumors, including breast, colorectal, and prostate cancer^23,26,37^. The cellular level of matriptase activation is well regulated by HAI-1 and the imbalance that favors matriptase contributes to cancer malignance^20,23^. In addition, the up-regulation of mariptase activation can be induced by ErbB-2 signaling^45^, COX-2^46^, and promoted by the other serine proteases, such as TMPRSS2 (Transmembrane protease, serine 2)^47^ and prostasin^48^.

Human HAI-2 (hepatocyte growth factor activator inhibitor-2), first identified in the placenta as a homology of HAI-1, has two Kunitz domains (KD1 and KD2) as well as a transmembrane region^49,50^. In addition to inhibiting HGFA^50^, HAI-2 is capable of repressing the proteolytic activities of many other human serine proteases, such as tissue kallikrein, plasma kallikrein, plasmin, and coagulation factor XIa^51^. Similar to HAI-1, HAI-2 is required for placental development^52^ and epithelial homeostasis^53^. Moreover, HAI-2 mutation has been found in congenital sodium diarrhea^54^ and congenital tufting enteropathy^55^. HAI-2, along with HAI-1, are mentioned together as tumor suppressors in ovarian cancer and uterine leiomyosarcoma^56,57^. A growing body of evidence further regards HAI-2 as a tumor suppressor, and its down-regulation is linked to poor prognosis in various cancers, including hepatocellular carcinoma, melanoma, esophageal squamous cell carcinoma, gastric cancer, renal cell carcinoma, prostate cancer, cervical cancer, medulloblastoma and ovarian cancer^58–68^. Furthermore, the potential anti-metastatic role of HAI-2 is demonstrated in hepatocellular carcinoma and melanoma^62,67^, and its KD1 is responsible for the inhibition of HGFA^69^ as well as cell invasion^62^.

Above all, our previous study indicates the pivotal role of HAI-2 in repressing matriptase activation, cell migration, invasion, tumorigenicity and metastasis of human prostate cancer^65^. However, although the down-regulation of HAI-2 is related to prostate cancer progression and matriptase activation^65^, the evidence and mechanism of HAI-2 regulating matriptase activation still remain elusive. Thus, the purpose of this study is to investigate whether HAI-2 is a cognate inhibitor of matriptase, and to identify which Kunitz domain of HAI-2 exerts its suppression function on matriptase proteolytic activity as well as prostate cancer cell invasion. We demonstrate that HAI-2 can physically interact with matriptase at the cell surface and repress matriptase proteolytic activity. Interestingly, although both recombinant KD1 and KD2 of HAI-2 have the ability to repress matriptase activity, cellular HAI-2 appears to only harness KD1 to repress matriptase activation and prostate cancer cell invasion. The results together indicate that HAI-2 is a cognate inhibitor of matriptase to suppress human prostate cancer cell invasion.

## Results

### HAI-2 down-regulation in the metastatic progression of prostate cancer cells

**Figure 1:**
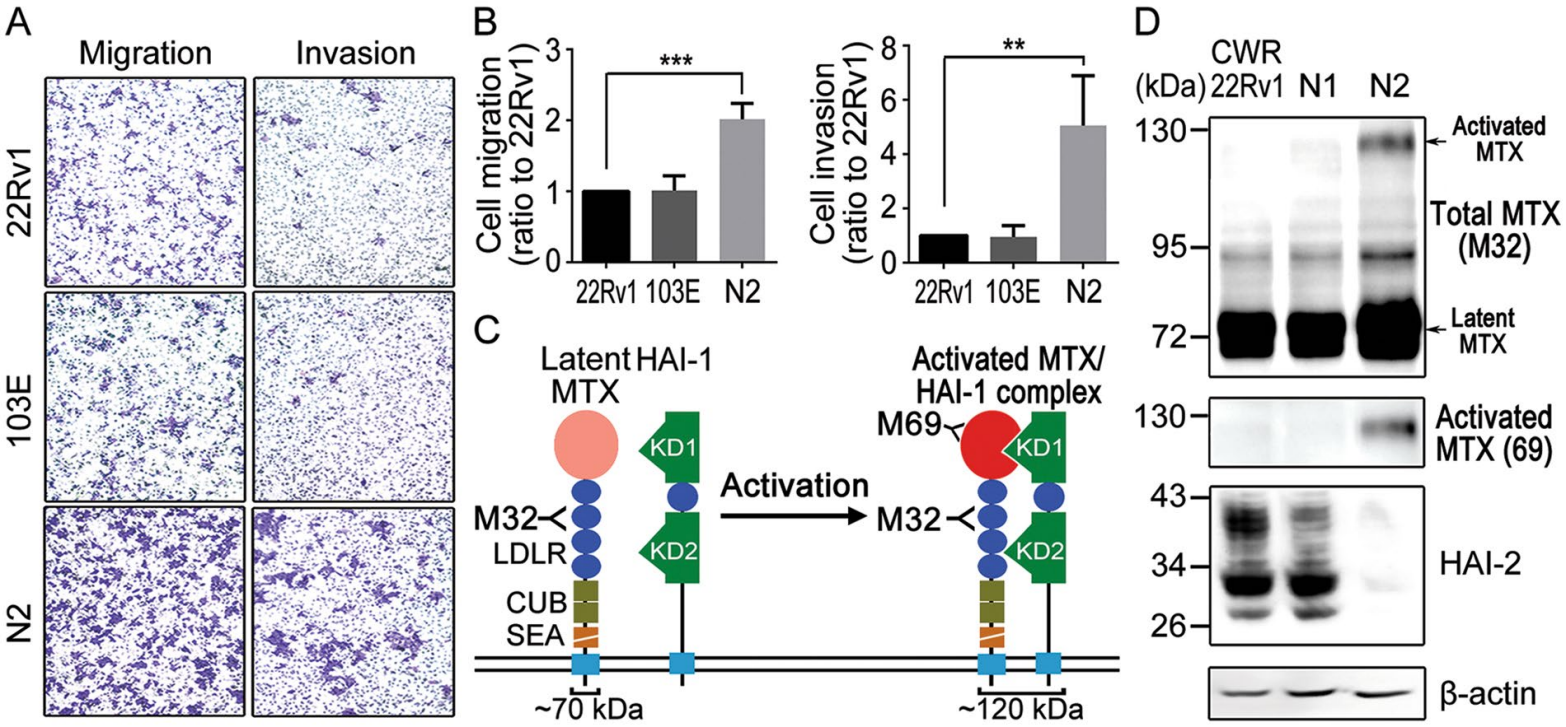
Role of HAI-2 in repressing matriptase activation and prostate cancer cell motility. (**A**) Examination of CWR22Rv1, 103E and N2 cell migration and invasion using transwell assays. Cells were seeded at a density of 2 × 10^5^ cells per well into a Boyden chamber coated with or without Matrigel. Regular culture medium containing 10% FBS was used as a chemoattractant in lower wells. The periods of incubation time were 24 hours for migration assays and 48 hours for invasion assays. The migrating and invasive cells on the bottoms of Boyden chamber were stained with 1% crystal violet and imaged by a microscopy (magnification, ×100). Three independent experiments were performed and a set of representative images were shown. (**B**) Quantification of the migrating and invasive cells in Fig. 1A by ImageJ software. The data were statistically calculated from of three independent experiments and presented as mean ± SD. (**p < 0.01; ***p < 0.001, one-way ANOVA). (**C**) The activation process of matriptase and the schematic structure of matriptase and HAI-1 on plasma membrane. Upon activation, latent matriptase (zymogen, 70 kDa) can undergo a cleavage process at the residue of R614 by autoactivation, androgen-induced TMPRSS2^47^, COX-2^99^ or ErbB-2 signaling^45^, to become active and immediately form a complex with HAI-1 (120 kDa). The activated matriptase-HAI-1 complexes were used to generate monoclonal antibodies and two monoclonal antibodies (M32 and M69) were used in this study. M32 can recognize the third LDLR domain of matriptase, and M69 can specifically interact with the activated protease domain of matriptase^100^, as shown in the figure. Matriptase is composed of an intracellular region in the amino terminus, followed by a transmembrane domain, one SEA, two CUB, four LDLR domains and a protease domain in the carboxyl terminus. HAI-1 has two Kunitz domains (KD1 and KD2) in the amino terminus, one LDLR domain between these two KDs, a transmembrane domain and a short intracellular region in the carboxyl terminus. Matriptase’s and HAI-1’s domain abbreviations: SEA, Sperm protein, Enterokinase and Agrin; CUB, C1r/C1s, Uegf and Bmp1; LDLR, LDL receptor^100^. (**D**) Immunoblot analysis of total and activated matriptase as well as HAI-2 in CWR22Rv1, 103E and N2 cells. Forty micrograms of cell lysate per sample were separated by SDS-PAGE and immunoblotted using monoclonal M32, monoclonal M69 and polyclonal anti-HAI-2 antibodies to detect total matriptase (activated and latent matriptase), activated matriptase, and HAI-2 proteins, respectively. β-actin was used as a control.

To show the relevance of HAI-2 with prostate cancer progression, we analyzed the expression levels of HAI-2 in a metastatic progression model of human prostate cancer CWR22Rv1 cells^65^. This model is briefly introduced as follows: The 103E cells (derivative cells from CWR22Rv1 cells carrying a luciferase reporter gene driven by a PSA promoter^65^) were implanted into the anterior prostates of mice. After 12 weeks, the metastatic cells in the lymph nodes near the injection site were isolated as N1 cells. The N1 cells underwent orthotropic injection and then the metastatic cells from the lymph nodes close to the injection site were denoted as N2 cells. The cell motilities (cell invasion and migration) of CWR22Rv1, 103E, and N2 cells were examined using transwell assays. The result showed that the cell motilities of 103E cells were similar to those of the parental CWR22Rv1 cells, while N2 cells obtained an increase of cell migratory and invasive capabilities by approximately 2 and 5 folds in comparison with 103E cells (Fig. 1A and B).

Our previous study showed that the down-regulation of HAI-2, following the metastatic progression of 103E cells, came with an increased level of activated matriptase^65^. Once matriptase activation occurs with a proteolytic cleavage on the residue of Arg614, it immediately forms a complex with HAI-1 with a molecular mass of 120 kDa^70^ (Fig. 1C). To detect total and activated levels of matriptase, we applied non-denatured, non-boiling western blot analysis using the monoclonal antibodies (mAbs) that can discriminate total or activated cellular matriptase^71^ (Fig. 1C). The M32 mAb detects the third LDLR domain of matriptase, including latent matriptase (70 kDa) and the activated matriptase-HAI-1 complex (120 kDa), while the M69 mAb specifically recognizes the activated matriptase protease domain of the matriptase-HAI-1 complex (120 kDa) (Fig. 1C). Because matriptase immediately binds to HAI-1 to form a 120-kDa complex upon activation, the presence of 120-kDa complex is a surrogate to indicate the activated level of matriptase. As shown in Fig. 1D, highly invasive N2 cells expressed a high level of activated matriptase and a low level of HAI-2, compared to CWR22Rv1 and 103E cells. The results illustrate an inverse correlation of HAI-2 and matriptase activation during prostate cancer metastatic progression.

### HAI-2 represses matriptase activation and matriptase-induced cell migration and invasion

**Figure 2:**
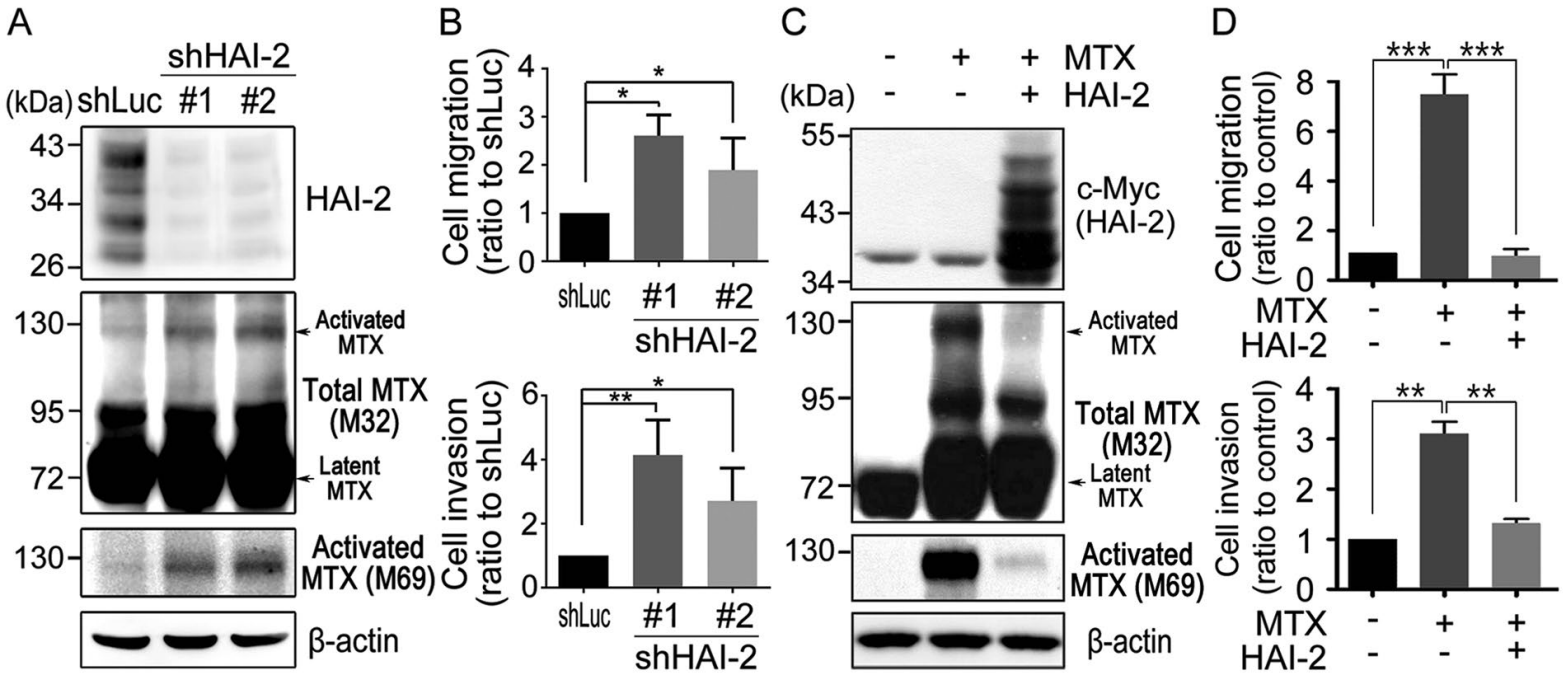
HAI-2 repressed matriptase activation and prostate cancer cell motility. (**A**) Effects of HAI-2 silencing on the total and activated matriptase in CWR22Rv1 cells. Cells were infected with lentiviral particles carrying the shRNAs against HAI-2 (shHAI-2 #1 and #2). A shRNA against luciferase (shLuc) was used as control. Forty micrograms of cell lysate per sample were taken for SDS-PAGE and analyzed by immunoblotting using polyclonal anti-HAI-2, monoclonal M32 and M69 Abs, respectively. β-actin was shown as control. (**B**) Analysis of HAI-2 knockdown effects on the CWR22Rv1 cell migration and invasion using transwell assays. Cells were seeded at a density of 2 × 10^5^ cells per upper chamber of Boyden chambers for cell migration (24 hours) and invasion assays (48 hours). The migrating and invasive cells on the bottoms of Boyden chambers were imaged, quantified by ImageJ, statistically calculated from three independent experiments and shown as mean ± SD. (*p < 0.05; **p < 0.01, one-way ANOVA). (**C**) Examination of the effects of matriptase and HAI-2 overexpression on prostate cancer cell migration and invasion. 103E cells were transfected with or without matriptase and HAI-2 plasmids. Stable pools of the transfectants with stable overexpression of matriptase or HAI-2 were indicated by “ + ”. “−” represented control with vector transfection. Forty micrograms of cell lysates per sample were subjected to SDS-PAGE and immunoblot analysis using anti-c-Myc, M32 and M69 mAbs to detect exogenous HAI-2, total matriptase and activated matriptase. (**D**) Analysis of the effects of the matriptase and HAI-2 overexpression on the cell migration and invasion by transwell assays. 103E cells were seeded at a density of 3 × 10^5^ cells per upper well of Boyden chambers and incubated for 20 hours. The data were statistically calculated from three independent experiments and presented as mean ± SD. (**p < 0.01; ***p < 0.001, one-way ANOVA).

To further examine the role of HAI-2 in matriptase activation, HAI-2 was genetically silenced by small hairpin RNAs. The result showed that reduced HAI-2 expression significantly enhanced the activated levels of matriptase (Fig. 2A) in CWR22Rv1 cells. Moreover, the knockdown of HAI-2 had no effect on the cell growth (Supplementary Figure S1), but significantly increased the cell migration and invasion (Fig. 2B and Supplementary Figure S2A). In the simultaneous overexpression of matriptase and HAI-2 in 103E cells, HAI-2 repressed the activated level of overexpressing matriptase (Fig. 2C), as well as matriptase-induced cell migration and invasion (Fig. 2D and Supplementary Figure S2B). The results together suggest that HAI-2 may function as a matriptase inhibitor, which is capable of inhibiting matriptase-elicited cell motility.

### HAI-2 interacts with matriptase on the cell surface and represses matriptase activity

**Figure 3:**
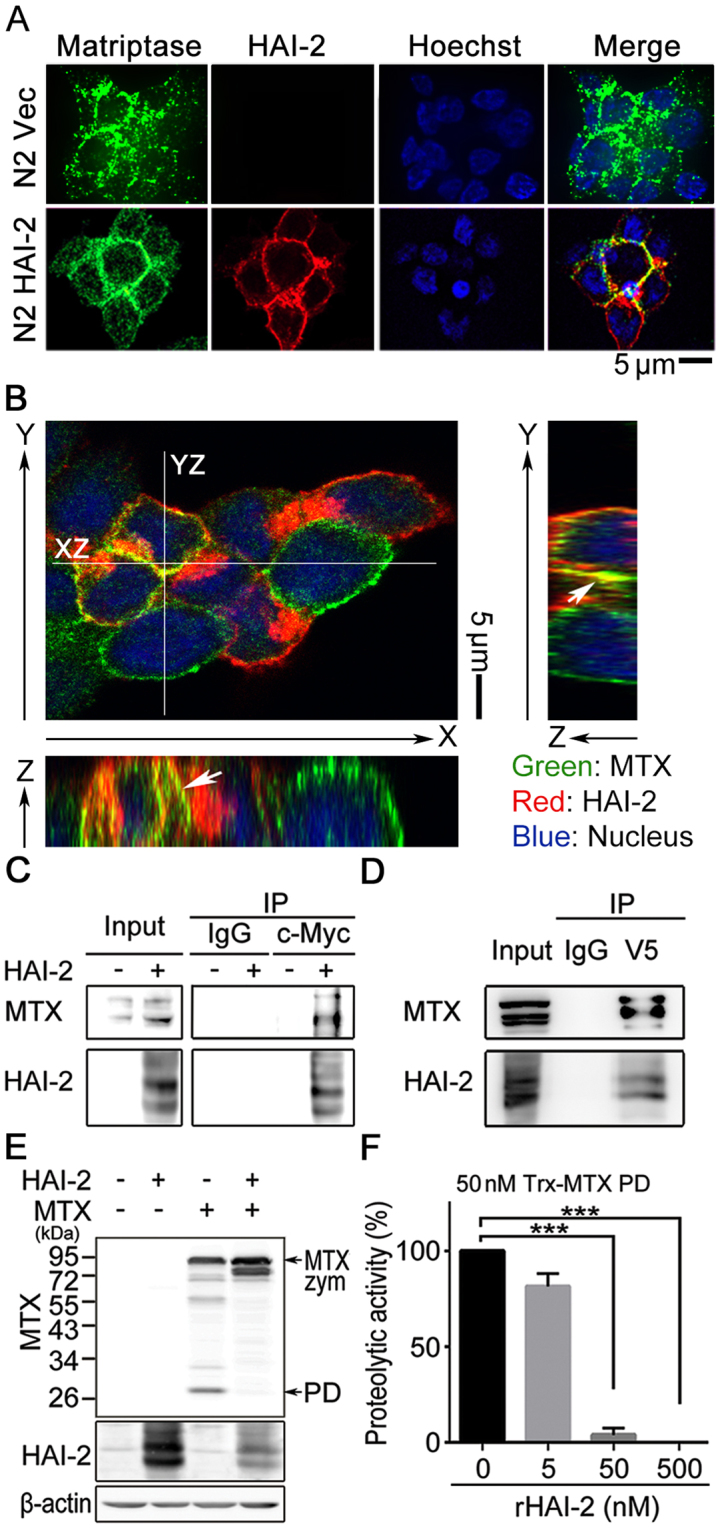
Subcellular localization and interaction of HAI-2 and matriptase in cells. (**A**) Fluorescent images of matriptase and HAI-2 in HAI-2-overexpressing N2 and N2 (Vec) cells. The subcellular localizations of HAI-2 and matriptase in HAI-2-overexpressing N2 cells were revealed by immunofluorescence and detected by a confocal microscope (Leica TCS SP5). Cellular matriptase was detected by M32 mAb as a primary Ab and fluorescein isothiocyanate-conjugated anti-mouse Ab as a secondary Ab (green). HAI-2 was revealed by a polyclonal anti-HAI-2 rabbit Ab (customized by Kelowna, Taipei, Taiwan) as a primary Ab and Rhodamine-conjugated anti-rabbit Ab as a secondary Ab (red). Nuclei were stained with Hoechst 33342 solution (blue, Excitation⁄Emission: 361⁄497 nm). (**B**) Fluorescent images of matriptase and HAI-2 in the Z axis of HAI-2-overexpressing N2 cells. Z-stack images (YZ and XZ) were generated by combining a series of images with incremental focuses (total 15 images) after the confocal microscope analysis. The arrows indicate the co-localization of matriptase and HAI-2. (**C**) Co-immunoprecipitation of matriptase and HAI-2 using a monoclonal anti-c-Myc Ab. Exogenous HAI-2 proteins with a c-Myc tag were immunoprecipitated by an anti-c-Myc mAb from the lysates of HAI-2-overexpressing N2 cells. Mouse IgG was used as control. The product after immunoprecipitation was subjected to SDS-PAGE and western blot analysis using anti-matriptase and anti-HAI-2 pAbs. (**D**) Co-immunoprecipitation of HAI-2 and matriptase using a monoclonal anti-V5 Ab. HEK293T cells were transiently transfected with matriptase (a V5 tag) and HAI-2 plasmids for both protein overexpression. The exogenous matriptase proteins were immunoprecipitated using an anti-V5 mAb from the cell lysates, and subjected to SDS-PAGE and western blot analysis using anti-V5 mAb (for matriptase) and anti-HAI-2 pAb. (**E**) Immunoblot analysis of matriptase and HAI-2 proteins in matriptase- or HAI-2-overexpressing HEK293T cells. HEK293T cells were transiently transfected to express matriptase or HAI-2 proteins. Cell lysates were collected for SDS-PAGE and immunoblot analysis using an anti-matriptase protease domain pAb (Cat. IM1014, Lot. 2733415, Millipore, CA, USA). Arrows were used to indicate the matriptase zymogen (MTX zym) and its protease domain (PD). HAI-2 protein levels were detected using an anti-HAI-2 pAb. β-actin was used as control. (**F**) Examination of the effects of purified recombinant HAI-2 (rHAI-2) proteins on the activity of matriptase using *in vitro* proteolytic assays. rHAI-2 proteins (Cat. 1106-PI-010, R&D system) were obtained for the *in vitro* proteolytic assays, which were originally produced from mouse NS0 cells and certified by the manufacture. The recombinant matriptase protease domain (Trx-MTX PD, 50 nM) was mixed with the indicated concentrations of purified rHAI-2 proteins (0, 5, 50 and 500 nM) in 50 μl PBS, pH 7.4. The mixtures were incubated at room temperature for 30 min. Fifty microliter of an artificial substrate (QAR-AMC, 100 μM) were then mixed with the sample solution (50 μl) in a well of 96-well black plates. The proteolytic activities of matriptase were revealed by measuring the increased intensity of fluorescence (EX: 360 nm; EM: 465 nm) after 1-hour reaction using a microplate reader (SpectraMax Paradigm, Molecular Device, CA, USA). The values of the proteolytic activity were statistically from three independent experiments, normalized to control and presented as mean ± SD. (n = 3, ***p < 0.001).

To examine whether HAI-2 could directly interact with matriptase, the subcellular localization and the interaction between both proteins were analyzed. The result from the immunofluorescent confocal microscopy illustrated that HAI-2 and matriptase were co-localized around plasma membrane in human prostate cancer HAI-2-overexpressing N2 cells, compared to N2 (Vec) cells (Fig. 3A). The Z-axis stack images further showed that HAI-2 and matriptase were co-localized in the cell-cell contact regions (Fig. 3B). These results together reveal that HAI-2 proteins appear at cell surface and are co-localized with matriptase at pericellular portions. The co-immunoprecipitation assays showed that matriptase was ably pulled down with HAI-2 in HAI-2-overexpressing N2 cells (Fig. 3C). The similar result from HEK293T cells also revealed that HAI-2 was captured in the immunoprecipitation of matriptase (Fig. 3D). In addition, matriptase overexpression underwent an auto-activation process with a release of its protease domain (Fig. 3E, third lane), while HAI-2 overexpression dramatically reduced the cleavage of protease domain (PD) (Fig. 3E, fourth lane). To further investigate whether HAI-2 exhibits a role in suppressing the proteolytic activity of matriptase, we performed an *in vitro* proteolytic activity assay, using the recombinant proteins of active matriptase protease domain (Trx-MTX PD, the protease domain of matriptase carrying the amino acid sequence from the residue 615 to 854 which was conjugated with an N-terminal thioredoxin tag) purified from an *E. coli* expression system (Supplementary Figure S3), an artificial substrate (Boc-Gln-Ala-Arg-AMC) for matriptase, and recombinant HAI-2 proteins purified from mouse myeloma cells. The results (Fig. 3F) showed that purified recombinant HAI-2 proteins significantly repressed the proteolytic activity of matriptase at an equal molar ratio (1:1) or higher. The data collectively indicates that HAI-2 binds to matriptase at cell surfaces, and inhibits matriptase activation and proteolytic activity.

### Recombinant HAI-2 proteins reduce matriptase activation and prostate cancer cell invasion

**Figure 4:**
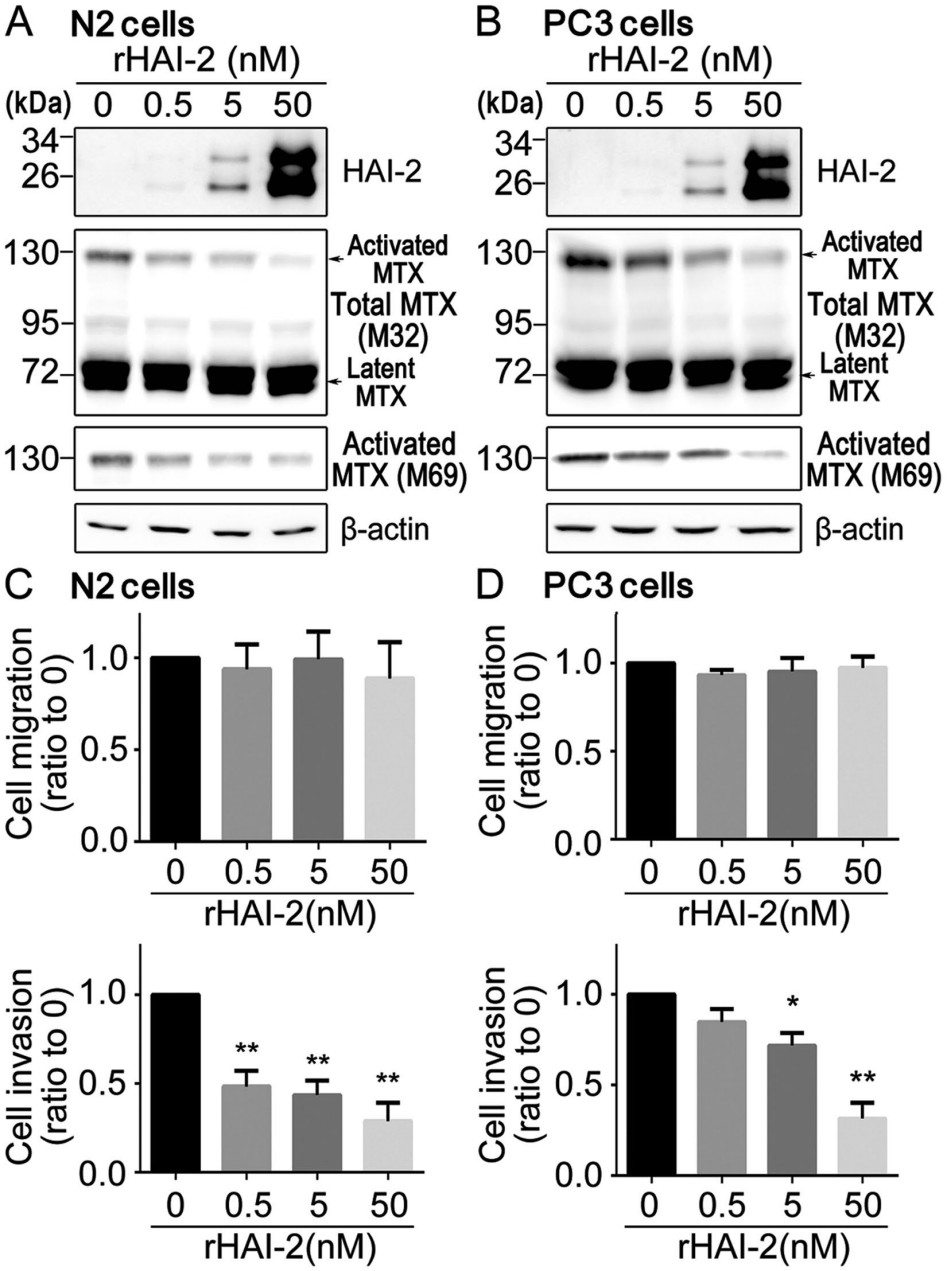
Purified recombinant HAI-2 proteins repressed cellular matriptase activation and prostate cancer cell motility. (**A**,**B**) Effects of purified recombinant HAI-2 proteins on matriptase in N2 (**A**) and PC-3 cells (**B**). Cells were treated with the indicated concentrations of rHAI-2 (Cat.1106-PI-010, R&D system, MN, USA, which was produced from mouse myeloma cells) and incubated for 20 hours. The conditioned media and cells lysates were collected for immunoblotting analysis using anti-HAI-2 pAb, M32 and M69 mAbs to detect the levels of rHAI-2 in the conditioned media and matriptase (total and activated levels) in cells, respectively. β-actin was used as control. (**C**,**D**) Effects of purified recombinant HAI-2 (rHAI-2) proteins on N2 (**C**) and PC-3 (**D**) cell migration and invasion. Cells were seeded at a density of 2 × 10^5^ (N2) or 5 × 10^4^ (PC-3) cells per well of Boyden chambers and treated with the indicated concentrations of rHAI-2 proteins. Boyden chambers were coated with or without Matrigel before cell seeding for cell invasion and migration assays. Migration assays were performed for 24 hours (N2) and 16 hours (PC-3) and invasion assays were carried out for 48 hours (N2) and 24 hours (PC-3). The results of cell migration and invasion were statistically calculated from three independent experiments and represented as mean ± SD. (*p < 0.05; **p < 0.01, one-way ANOVA).

Since the above results indicate that HAI-2 may mainly exert its anti-proteolytic function on the cell surface, we further explored if the addition of recombinant HAI-2 (rHAI-2) proteins could repress matriptase activation, prostate cancer cell invasion and migration. The data illustrated that the rHAI-2 proteins effectively inhibited matriptase activation (Fig. 4A and B), as well as the cell invasion of N2 and PC-3 cells in a dose-response manner (Fig. 4C and D, lower panels; Supplementary Figure S4). Unexpectedly, rHAI-2 proteins had no significant effect on the cell migration (Fig. 4C and D, upper panels; Supplementary Figure S4). These results indicate that purified recombinant HAI-2 proteins can suppress matriptase activation and prostate cancer cell invasion.

### Inhibitory effects of recombinant HAI-2 KD1 and KD2 proteins on the *in vitro* proteolytic activity of matriptase

**Figure 5:**
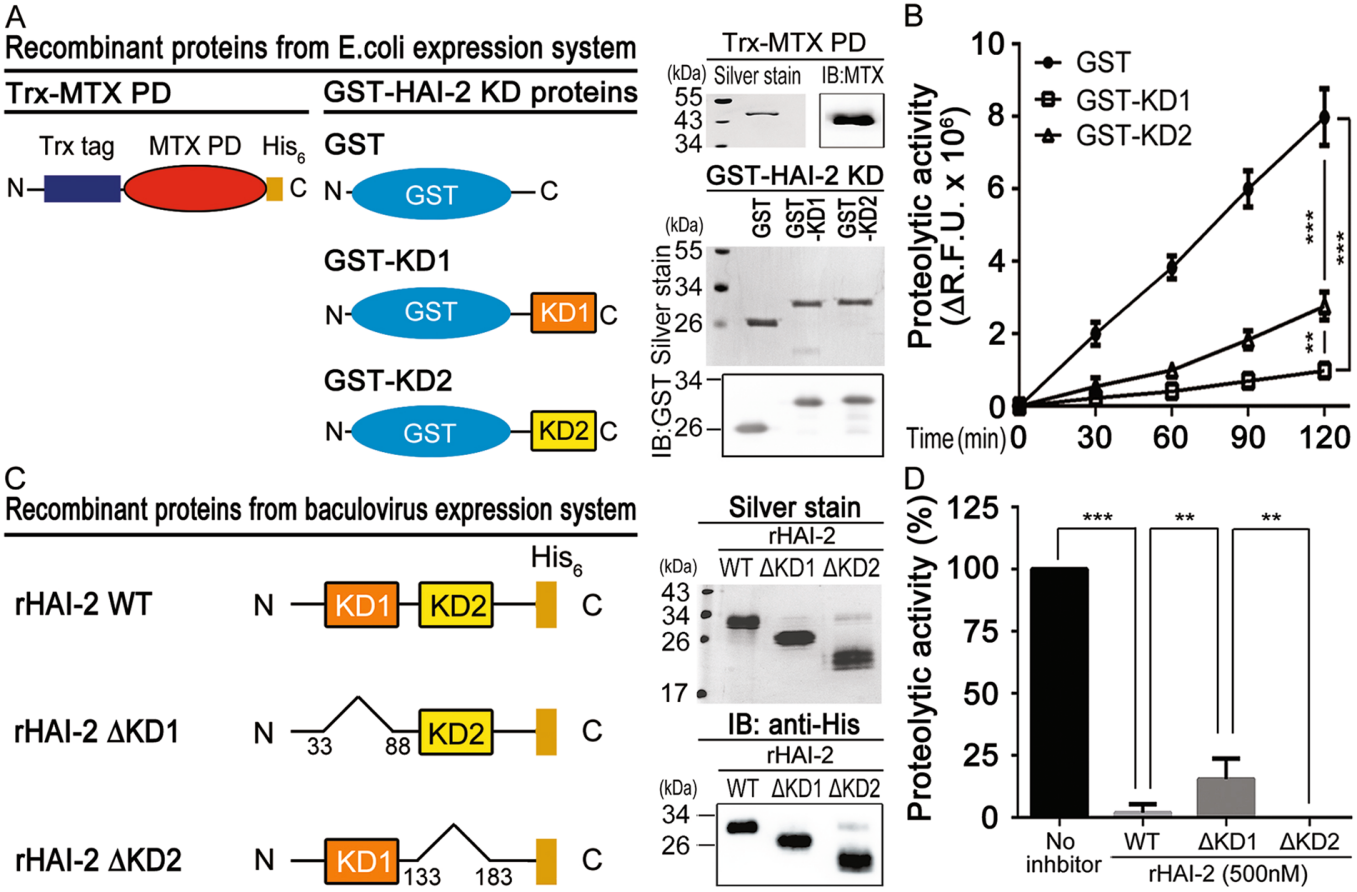
Inhibitory effects of HAI-2’s KD1 and KD2 on the *in vitro* proteolytic activity of matriptase. (**A**) The schematic structures and protein purification of recombinant matriptase’s protease domain (Trx-MTX PD) and GST HAI-2 KD fusion proteins. The fusion proteins were purified from *E. coli* expression system (Detailed information described in the Materials and Methods). The purified fusion proteins (Trx-MTX PD and GST-HAI-2 KDs) were subjected to SDS-PAGE, silver staining and immunoblotting using anti-matriptase pAb and anti-GST mAb, respectively. (**B**) Examination of the inhibitory effects of purified GST-HAI-2 KD1 and GST-HAI-2 KD2 proteins on the *in vitro* proteolytic activity of matriptase (Trx-MTX PD). For the protease activity assays, each purified HAI-2’s KD-fusion proteins (GST, GST-KD1 and GST-KD2, 500 nM) was gently mixed with Trx-MTX PD fusion proteins (100 nM) in 50 μl PBS and incubated for 30 min. Fifty microliters of an artificial substrate (QAR-AMC, 100 μM) were then added into the above solution and gently mixed in a well of 96-well black plate. The intensity of fluorescence (EX: 360 nm; EM: 465 nm) after the proteolytic reaction was recorded using a microplate reader in an interval of 30 min for 2 hours (SpectraMax Paradigm, Molecular Device, CA, USA). The effects of purified HAI-2- KD GST-fusion proteins on the proteolytic activity of matriptase were represented by the change of relative fluorescence units (ΔR.F.U.). The results were statistically calculated from three independent experiments and shown as mean ± SD. (**p < 0.01; ***p < 0.001, one-way ANOVA). (**C**) The schematic structures of rHAI-2 and its KD-deleted mutants (rHAI-2ΔKD1with KD1 deletion; rHAI-2ΔKD2 with KD2 deletion) and those fusion proteins purified from a baculovirus expression system. The recombinant HAI-2 fusion proteins were purified from a baculovirus expression system (Detailed information described as in the Materials and Methods), and then subjected to SDS-PAGE, silver staining and immunoblotting using an anti-His mAb to reveal the purified rHAI-2 and mutant proteins (right panel). (**D**) Examination of the inhibitory effects of purified rHAI-2 proteins (WT and its domain-deleted mutants) on matriptase’s proteolytic activity. The solution of purified rHAI-2 (WT) and its KD-deleted mutant proteins (rHAI-2ΔKD1 and rHAI-2ΔKD2) (500 nM per sample) were gently mixed with the solution of Trx-MTX PD proteins (100 nM) in 50 μl PBS and incubated for 30 min. Fifty microliter of the substrate (QAR-AMC, 100 μM) were then added into the above solution and mixed well in a well of 96-well black plates. The increased levels of fluorescence (EX:360 nm; EM:465 nm) after 1-hour reaction were recorded using a microplate reader (SpectraMax Paradigm, Beckman Coulter, CA, USA). The results were statistically calculated from three independent experiments and represented as mean ± S.D with normalization to no inhibitor control. (n = 3; **p < 0.01).

Next, we investigated if the KD1 and KD2 of HAI-2 exhibited a differentially inhibitory effect on the proteolytic activity of matriptase by *in vitro* enzymatic assays. First, we purified the recombinant fusion proteins of matriptase protease domain (Trx-MTX PD), HAI-2’s KD1 and KD2 with an N-terminal GST tag (GST-KD1 and GST-KD2) from an *E. coli* expression system [Fig. 5A; Supplementary Figure S3 (Trx-MTX PD), S5A and S5B (GST-KDs)], and assessed the effects of HAI-2’s KDs on the proteolytic activities of matriptase by using an artificial substrate (Boc-Gln-Ala-Arg-AMC). The results showed that both GST-KD1 and GST-KD2 were able to inhibit matriptase, and the inhibitory efficiency of GST-KD1 was better than that of the GST-KD2 (Fig. 5B). In addition, we generated and purified the recombinant HAI-2 (rHAI-2) proteins [wild type (WT), KD1-deleted mutant (ΔKD1) and KD2-deleted mutant (ΔKD2)] from a baculovirus vector expression system (Fig. 5C; Supplementary Figure S5C and S5D). Similarly, the result of the proteolytic activity assays (Fig. 5D) manifested that all these rHAI-2 proteins were able to repress matriptase. Among them, the inhibitory potency of rHAI-2 WT and rHAI-2 ΔKD2 proteins on the matriptase activity reached up to 99%, with a higher efficacy than that of rHAI-2 ΔKD1 (~80%) (Fig. 5D). The results together indicate that both HAI-2’s KD1 and KD2 are capable of repressing the proteolytic activity of matriptase protease domain.

### Dynamic bindings and computational docking models of HAI-2’s KD1 and KD2 with matriptase protease domain

**Figure 6:**
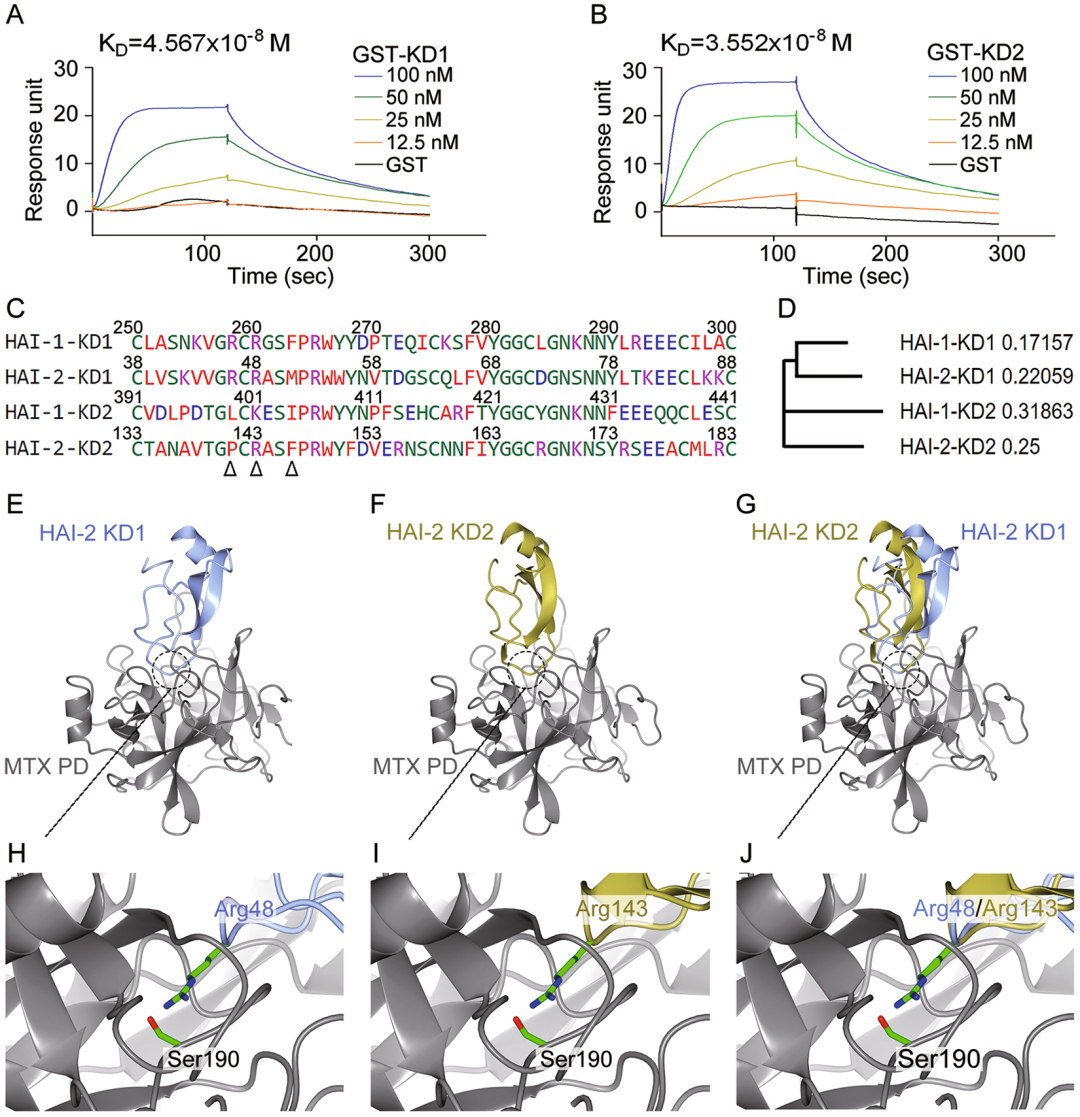
Dynamic bindings and computational molecular docking between HAI-2’s KDs and matriptase’s protease domain. (**A**,**B**) Examination of the dynamic bindings of GST-KD1 (**A**) and GST-KD2 (**B**) to matriptase’s protease domain using BIAcore assay. The purified recombinant HAI-2’s KD1 and KD2 GST-fusion proteins were dialyzed and diluted in running buffer to the indicated concentrations. One hundred nanomolar of GST fusion proteins were used as a negative control. To measure the dynamic bindings between HAI-2’s KDs and matriptase’s protease domain, the indicated concentrations of the analytes (HAI-2’s KD GST-fusion proteins) were injected to Trx-MTX PD-immobilized CM5 chip at 30 µl/min for 120 sec. The dissociation was carried out by passing running buffer 30 µl/min for 180 sec. The estimated dissociation constants (K_D_) were statistically calculated by the affinity analysis of BIAevaluation software v1.0 (GE Lifescience, CT, USA). (**C**) The amino acid sequence alignment of the HAI-1’s and HAI-2’s KD1 and KD2 functional regions. The amino acid sequence alignment was performed by the MUltiple Sequence Comparison by Log-Expectation (MUSCLE, EMBL-EBI). Δ indicated the important residues which potentially interacted with matriptase’s protease domain^40^. (**D**) The neighbor-joining tree of phylogenetic analysis was performed by MUSCLE (EMBL-EBI). The values mean the genetic distances for each KD. (**E**,**F**) The computational docking models of HAI-2 KD1 (**E**) and KD2 (**F**) with matriptase’s protease domain. The structural models of matriptase’s protease domain (Matriptase PD, PDB ID: 4ISO) and HAI-2 KD1 (PDB ID: 4U32) were downloaded from Protein Data Bank. HAI-2’s KD2 was constructed by the computational docking according to HAI-2’s KD1 (PDB ID: 4U32). The best-fit docking models of HAI-2’s KD1-matriptase PD and HAI-2’s KD2-matriptase PD were generated by ClusPro 2.0 (https://cluspro.bu.edu/)^77–81^ and drew by CCP4mg software (MRC Laboratory of Molecular Biology, Cambridge, UK). (**G**) The superposition of the docking models of KD1 and KD2 with matritpase’s protease domain. (H/I) The interaction residues between the active site in HAI-2’s KD1 (R48) (**H**) and KD2 (R143) (**I**) and matriptase’s protease domain (catalytic site S190) in the docking models. (**J**) The superposition of the residue R48 in HAI-2’s KD1 and the residue R143 in HAI-2’s KD2 with the residue S190 in matriptase’s protease domain in the docking models.

To further understand the interaction between each HAI-2’s KD (KD1 or KD2) and matriptase’s protease domain, we performed SPR (surface plasmon resonance)-based BIAcore assay to study the dynamic bindings of KDs to matriptase protease domain^72,73^. The results showed that both KD1 and KD2 of HAI-2 had strong affinities with matriptase’s protease domain, because of their dissociation constants at the range of 10^−8^ M (Fig. 6A and B). To explain how HAI-2’s KD1 and KD2 interact with matriptase’s protease domain, we analyzed the amino acid sequences and protein structures of HAI-2 KDs aligned with HAI-1’s KD1 and KD2, since it has been shown that HAI-1 inhibits matriptase activity *via* its KD1^39^, and the key residues of HAI-1’s KD1 in contact with matriptase’s protease domain are R258, R260 (active site) and F263^40^ (Fig. 6C, marked by Δ). The homologous alignment of HAI-1’s and HAI-2’s KDs showed that the correspondent key residues in HAI-2 were R46, R48 (active site) and M51 in HAI-2’s KD1; P141, R143 (active site) and F146 in HAI-2’s KD2 (Fig. 6C). Although each KD of HAI-2 has a substitute of the key residue (HAI-2 KD1’s M51 in place of HAI-1 KD1’s F263; HAI-2 KD2’s P141 in place of HAI-1 KD1’s R258), these substitute residues do not significantly interfere the interaction with matriptase protease domain. Moreover, the phylogenetic analysis from the alignment indicates that HAI-1’s KD1 and HAI-2’s KD1 have a more homologous relationship (Fig. 6D).

Furthermore, we performed the computational docking of HAI-2’s KD1 and KD2 with matriptase protease domain using the crystal structures (HAI-2’s KD1, PDB ID: 4U32^74^; matriptase’s protease domain, PBD ID: 4ISO^40^; the model of HAI-2’s KD2 was constructed by SWISS-MODEL^75,76^ using HAI-2’s KD1 as a template) and ClusPro web server^77–81^. The predicted docking models showed that both KD1 and KD2 of HAI-2 had close contacts with the protease domain of matriptase (Fig. 6E and F), although their binding orientations were different (Fig. 6G). Moreover, both active sites (R48 for KD1 and R143 for KD2) of HAI-2’s KD1 and KD2 deeply insert into the protease domain and directly contact with the catalytic residue (S190) of matriptase (Fig. 6H–J). The results of dynamic bindings and docking prediction indicate that both KD1 and KD2 of HAI-2 can interact with the protease domain of matriptase with a direct contact between KDs’ active sites and matriptase’s catalytic residue.

### Kunitz domain 1 of HAI-2 responsible for its inhibitory effect on cell migration and invasion

**Figure 7:**
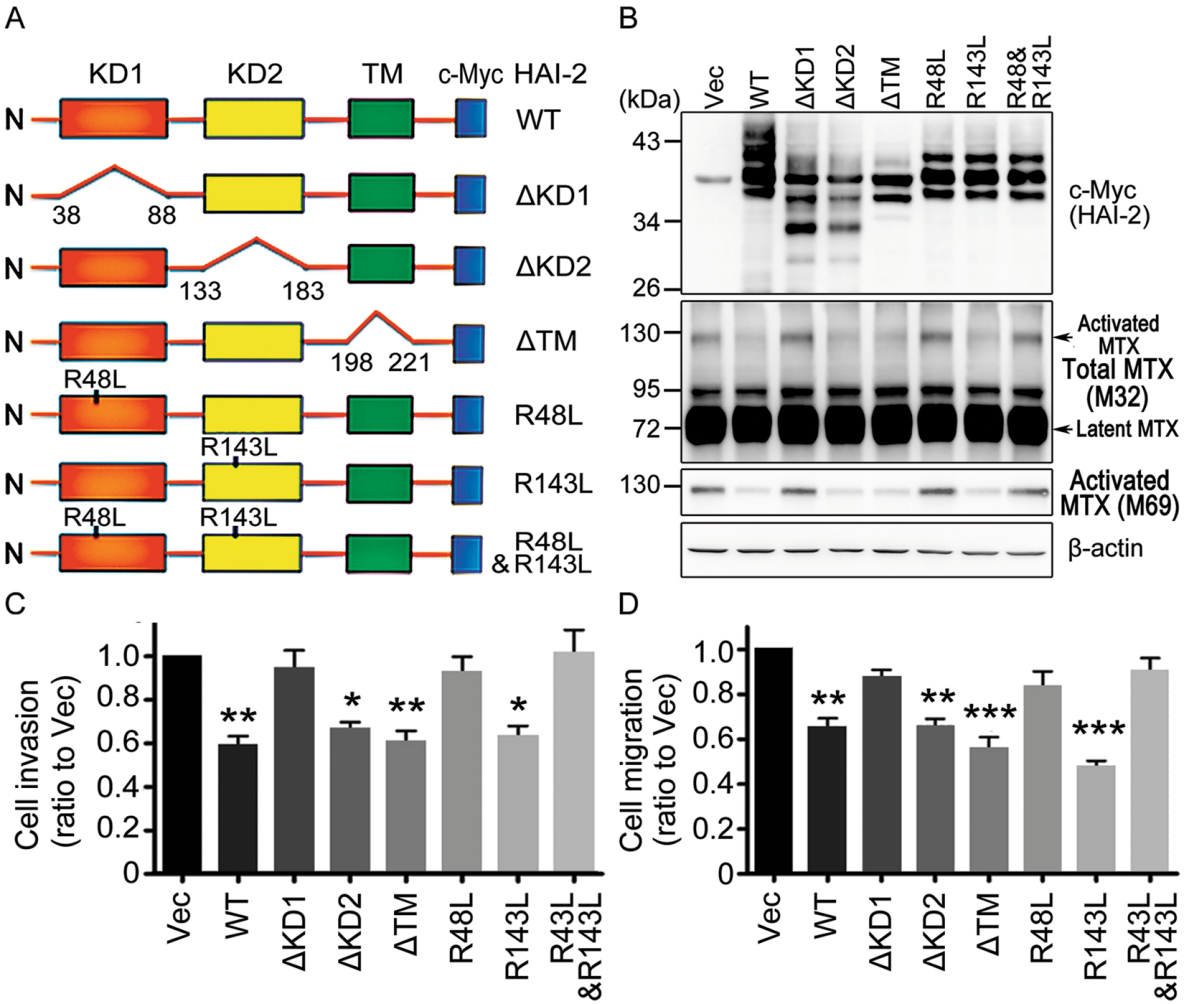
Characterization of HAI-2’s KDs in inhibiting matriptase activation and prostate cancer cell motility. (**A**) Schematic structures of wild-type HAI-2 and its variant mutants with domain deletions or active site mutations. (**B**) N2 cells were stably transfected with the plasmids encoding the wild type and mutants of HAI-2. Western blot revealed the levels of HAI-2 protein, total matriptase and activated matriptase using anti-c-Myc, M32 and M69 mAbs, respectively. β-actin was shown as control. (C/D) Examination of the effects of HAI-2 mutant proteins on N2 cell invasion (**C**) and migration (**D**) using transwell assays. N2 cells which stably expressed HAI-2 mutant proteins were seeded at a density of 3 × 10^5^ cells per upper chamber of Boyden chambers and incubated for 16 hours. Data were statistically calculated from three independent experiments and shown as mean ± SD. (*p < 0.5; **p < 0.01; ***p < 0.001, one-way ANOVA).

To characterize the *in vivo* functions of HAI-2’s KD1 and KD2 in the regulation of cellular matriptase activity and prostate cancer cell motility, we constructed domain-deleted and activity-null mutants of HAI-2 (Fig. 7A). The active sites of HAI-2’ KDs, R48 (KD1) and R143 (KD2) (Fig. 6), were replaced with Leu to abolish the activity of HAI-2’s KD1 and KD2^82^. After transfection and antibiotic selection, we established stable pools of N2 cells bearing these variant HAI-2 mutants. The results from immunoblotting show that the HAI-2 mutants with a deletion on KD1 (ΔKD1) or mutations [R48L and double mutation (DM, R48L/R143L)] lose HAI-2’s inhibitory ability on matriptase activation (Fig. 7B). In comparison with wild-type HAI-2, the HAI-2 KD1 deletion (ΔKD1), KD1 mutation (R48L) and double mutation (R48L/R143L) show no significant effects on the cell migration and invasion (Fig. 7C and D; Supplementary Figure S6). The HAI-2 mutants with a deletion of KD2 (ΔKD2), transmembrane domain-deletion (ΔTM) and KD2 mutation (R143L), still could significantly decrease the cell migratory and invasive abilities, similar to wild-type HAI-2 (WT) (Fig. 7C and D; Supplementary Figure S6). Together, these results show that KD1 of HAI-2 is a key domain to inhibit cellular matriptase activation and prostate cancer cell motility.

### Recombinant HAI-2 proteins containing Kunitz domain 1 repressed matriptase activation and prostate cancer cell invasion

**Figure 8:**
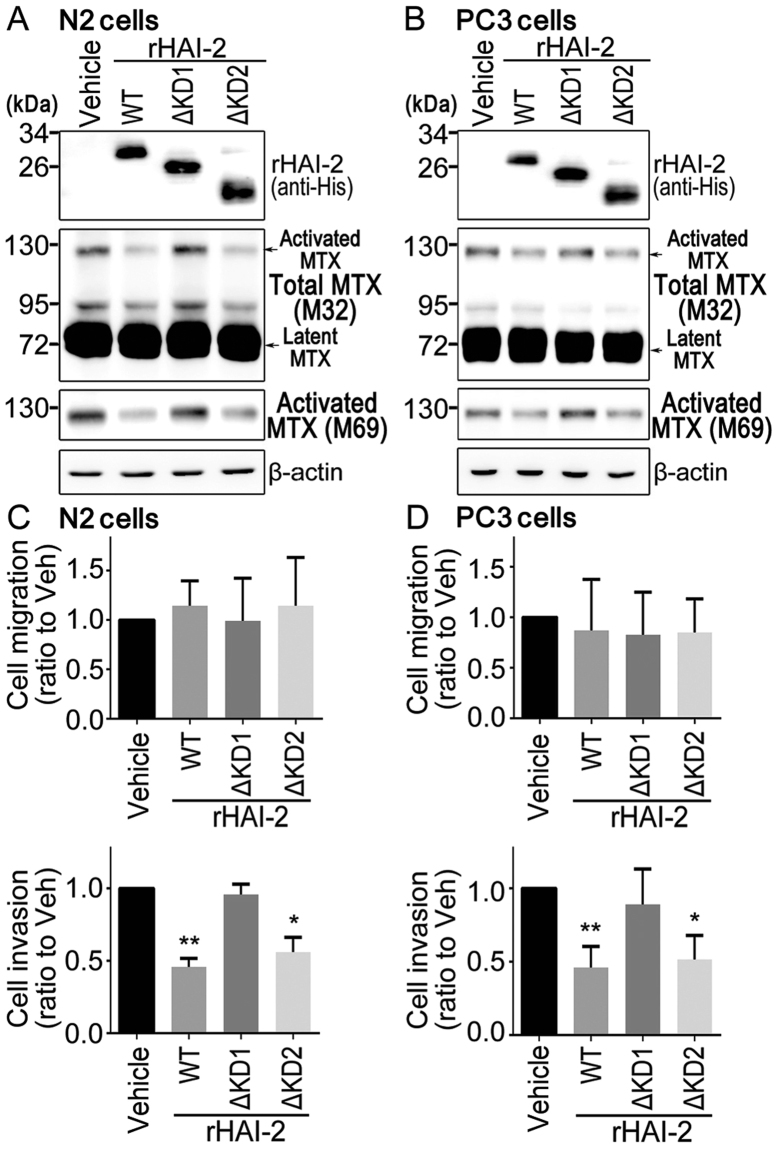
Recombinant HAI-2 proteins containing KD1 inhibited matriptase activation and prostate cancer cell invasion. (**A**,**B**) Examination of the effects of purified recombinant HAI-2 and its domain-deleted mutant proteins on matriptase activation in N2 (**A**) and PC-3 cells (**B**) using immunoblotting. Cells were then treated with 50 nM of recombinant HAI-2 proteins [wild-type (WT), HAI-2 mutant proteins with KD deletion (ΔKD1 and ΔKD2)] and incubated for 20 hours. The conditioned media and cells lysate were collected for Western blot using anti-His (HIS.H8, Cat. MA1-21315, Lot. RA225460, Thermo Fisher, MA), M32 and M69 mAbs to detect the levels of rHAI-2 in conditioned media and matriptase (total matriptase and activated matriptase) in cell lysates, respectively. β-actin was used as control. (**C**,**D**) Analysis of the effects of purified recombinant HAI-2 proteins with domain deletion on the migration and invasion of N2 (**C**) and PC-3 cells (**D**) using transwell assays. Cells were seeded at a density of 2 × 10^5^ (N2) and 5 × 10^4^ (PC-3) cells per well of Boyden chambers and treated with 50 nM of purified wild-type (WT) and domain-deleted mutants (ΔKD1 and ΔKD2) of rHAI-2. Migration assays were performed for 24 hours (N2) and 16 hours (PC-3), and invasion assays were carried out for 48 hours (N2) and 24 hours (PC-3). Data were statistically calculated from three independent experiments and represented as mean ± SD. (*p < 0.5; **p < 0.01, one-way ANOVA).

To assess the effects of rHAI-2’s KDs on cellular matriptase activation and prostate cancer cell motility, we utilized the recombinant variant HAI-2 proteins which were purified from baculovirus vector expression system (Fig. 5C) to treat prostate cancer cells. As shown in Fig. 8A and B, the rHAI-2ΔKD1 proteins lose their inhibitory activity against matriptase activation, while the rHAI-2ΔKD2 proteins still keep an inhibitory function on matriptase in N2 and PC-3 cells, similar to wild-type rHAI-2 proteins. Likewise, rHAI-2ΔKD1 proteins are unable to repress the invasion of N2 and PC-3 cells, whereas rHAI-2ΔKD2 proteins have a suppression effect on the cell invasion similar to wild-type rHAI-2 (Fig. 8C,D and Supplementary Figure S7). Similar to the above results (Fig. 4C and D), these recombinant HAI-2 variant proteins have no significant effect on the cell migration (Fig. 8C,D and Supplementary Figure S7). The data together indicates that KD1 is a crucial region of the rHAI-2 protein to down-regulate matritpase activation and prostate cancer cell invasion.

## Discussion

The imbalance of proteases and their cognate inhibitors often causes dysregulation of proteolysis and results in several disease development and progressions, including cancer metastasis^1–3^. Among them, matriptase and HAI-2 were intensely investigated because of their roles in tumorigenicity and cancer progression^8,22,24,45,47,63^. Although the data from the previous study suggests that HAI-2 has an inhibitory role in matriptase-elicited proteolytic cascade^65^, there is a lack of direct evidence to demonstrate HAI-2 as a cognate inhibitor of matriptase in human prostate cancer. By exploring the evidence further in this study, our results show that HAI-2 interacts with matriptase on plasma membrane and directly inhibits matriptase’s proteolytic activity, autoactivation, as well as prostate cancer cell invasion. Moreover, we found that cellular HAI-2 can bind with matriptase to form a 95-kDa complex in the absence of HAI-1 (Supplementary Figure S8A), but only can reduce the levels of HAI-1-matriptase complex in the presence of HAI-1 (Supplementary Figure S8B). These results together strongly indicate that HAI-2 serves as a cognate inhibitor of matriptase in human prostate cancer cells.

Previous studies have indicated the important role of HAI-2’s KD1 in preventing matriptase shedding^82^. A recent investigation revealed that HAI-2’s KD1 mutations (C47F and R48L) reduced the activity of HAI-2 to repress matriptase^83^. Our data further illustrates the functional role of HAI-2’s KD1 in repressing matriptase activation as well as prostate cancer cell motility. These findings together manifest that HAI-2’s KD1 is a key domain responsible for the regulation of matriptase.

Although both recombinant KD1 and KD2 of HAI-2 were able to inhibit and interact with matriptase in the *in vitro* study (Figs 5 and 6), cellular HAI-2 tended to harness its KD1 to represses matriptase activation as well as prostate cancer cell motility, rather than its KD2 (Figs 7 and 8). An interpretation is that the molecular structure of membrane-anchored matriptase limits HAI-2’s KD2 but favors KD1 to access the protease domain and restrains its KD2’s function. Considering the different docking poses of HAI-2’s KD1, KD2 (Fig. 6G) and the little flexibility of membrane-anchored matriptase, it is plausible that the molecular hindrance and rigidity may permit KD1 docking but impede KD2 binding. In the enzymatic experiments (Fig. 5), on the other hand, the recombinant matriptase protease domain which lacks the stem region may lose the structural hindrance for HAI-2’s KD2, such that the recombinant proteins of HAI-2’s KD2 could interact with matriptase’s protease domain and inhibit the proteolytic activity. In spite of the inhibitory function of each HAI-2’s KD on matriptase’s protease domain, cellular HAI-2 to suppress matriptase and human prostate cancer cell motility is mainly *via* its KD1.

HAI-2’s KD1 has also been identified as an important region to inhibit the proteolytic activity of HGFA^69^. Nevertheless, a recent study has shown that a mutation of T163C at HAI-2’s KD2 is involved in congenital sodium diarrhea and results in loss of function against TMPRSS13 and prostasin activities^84^. Similar to the role of HAI-2 in HGFA, our current results indicate that HAI-2’s KD1 has a significant role in inhibiting matriptase and prostate cancer cell invasion. These findings together point out that the KD1 and KD2 domains of HAI-2 have individual roles in modulating respective target proteases. On the other hand, the KD1 and KD2 of HAI-2 may also play differential roles among species. Several lines of evidence have shown that human HAI-2 employs its KD1 to repress HGFA^62,69^, while the KD2 of HAI-2 is a critical region to suppress HGFA activity in mice^85^. Moreover, the truncated HAI-2 proteins only bearing KD2 are found to be predominantly expressed in mouse tissues^86^. These findings indicate that mouse HAI-2 mainly employs its KD2 to exert its biological functions. It raises an interesting question of which domain is important for HAI-2 to inhibit mouse matriptase, although our current results support that the KD1 of HAI-2 is a main region for HAI-2 to inhibit human matriptase in prostate cancer cells.

HAI-1 and HAI-2 both have two Kunitz domains (KD1 and KD2)^69,87^, and their KD1s are homologous (Fig. 6D), implying that HAI-1’s and HAI-2’s KD1s may share similar biological functions. The *in vitro* protease enzymatic assay shows that the KD1 of HAI-1 plays a major role in repressing HGFA^87^ and matriptase^39^, as well as anti-invasive ability against human glioblastoma cells^88^. For cancer therapy, PEGylated KD1 proteins derived from HAI-1 have been developed to suppress prostate tumor growth and metastasis through inhibiting hepsin^89^. Similar to the crucial role of HAI-1’s KD1 on HGFA and hepsin, we found that HAI-2’s KD1 exhibits a potent inhibition on matriptase activity and prostate cancer cell invasion. Since it has been suggested that HAI-2 serves as a more effective inhibitor on matriptase than HAI-1^90^, our results support that HAI-2’s KD1 may have a therapeutic potential to be a potent inhibitor for matriptase and prostate cancer invasive growth.

HAI-2 is proven to be a highly glycosylated protein (Supplementary Figure S9 and ref.^91^). The immunoblotting pattern of the rHAI-2 proteins which were expressed and purified from mouse cells (Fig. 4A and B) shows two major bands (~26 and 30 kDa) which also appears in a similar pattern in human prostate cancer cells (Figs 1D and 2A). For insect cells, however, the expression pattern of rHAI-2 is quite different, showing one major band with a molecular mass of 30 kDa (Figs 5D and 8A,B). The results indicate that rHAI-2 expressed in different species may alter its glycosylation patterns. Nevertheless, no matter what expression systems (mouse, insect cells or *E. coli*) rHAI-2 proteins are obtained from, all of them have inhibitory effects on matriptase activity. As a result, the glycosylation might contribute little to HAI-2 functions to inhibit matriptase and prostate cancer cell motility.

The results showed that the overexpression of HAI-2 suppresses the migration and invasion of human prostate cancer cells (Figs 2 and 7), while the treatment of purified recombinant HAI-2 (rHAI-2) proteins is only able to repress prostate cell invasion without any effect on migration (Figs 4C,D and 8C,D). Moreover, the overexpression of HAI-2ΔTM plasmids (sharing an identical amino acid sequence with rHAI-2) is able to repress both cell migration and invasion (Fig. 7). The different effects of the same extracellular HAI-2 portion from the overexpression (HAI-2 ΔTM) and the purification (rHAI-2) on the prostate cancer cell migration may be due to the procedure of protein intracellular trafficking. The HAI-2 proteins that were expressed from HAI-2ΔTM plasmids through intracellular trafficking may modulate some intracellular target(s) to block cell migration machinery, while the rHAI-2 proteins have no such effect because of the inaccessibility to these intracellular target(s). Whether HAI-2 has intracellular target(s) to modulate cell migration remains unclear and is worthy of further investigation. For HAI-2 to inhibit cell invasion, on the other hand, there are other targets in the pericellular region which are accessible to rHAI-2 proteins. In this study, we demonstrate that HAI-2 proteins also exist on the cell surface (Supplementary Figures S10 and S11) in addition to being an intracellular protein, and identify matriptase as one of the HAI-2’s pericellular targets in human prostate cancer cells. Extensively, there may be an additional target for HAI-2 to suppress prostate cancer cell invasion. One of the potential proteases is a GPI-anchored serine protease prostasin, because it has been shown that HAI-2 is an inhibitor of prostasin and HAI-2 silencing leads to matriptase activation and shedding caused by prostasin in colon cancer cells and mouse models^92,93^. Since prostasin is also expressed in human prostate cancer cells^94^, one of the alternative pathways for HAI-2 down-regulation to cause matriptase activation in prostate cancer cells might be due to the increased prostasin activity. On the other hand, prostasin has been reported to be a tumor suppressor^94^ and its expression is down-regulated in highly invasive prostate cancer cells^95^. Thus, for prostate cancer, whether prostasin is involved in HAI-2-modulated matriptase activation is still elusive and needs more investigations. Moreover, it remains an interesting question and worthy of further studies whether the down-regulation of HAI-2 could indirectly induce matriptase activation through a novel protease. Nevertheless, our current data reveals that HAI-2 can directly inhibit matriptase’s proteolytic activity and activation in human prostate cancer cells.

In conclusion, our current study indicates that HAI-2 is a cognate inhibitor of matriptase in human prostate cancer cells and employs its KD1 to inhibit matriptase proteolytic activity as well as prostate cancer cell invasion. The results tentatively suggest that the KD1 of HAI-2 exhibits potential against invasive tumor growth and metastasis of human prostate cancer.

## Material and Methods

### Cell culture

Human prostate cancer CWR22Rv1, 103E and N2 cells were provided by Dr. Pen-Wei Hsiao at the Center of Agricultural Biotechnology, Academia Sinica, Taiwan and maintained in RPMI 1640 (Cat. 318000; Lot. 1786045, Thermo Fisher, MA, USA) supplemented with 10% FBS (Cat. SH30071.03; Lot. AZB182961, Hyclone, IL, USA) and 1% L-glutamine (Cat. G8540, Lot. SLBN0406V, Sigma-Aldrich, MO, USA). Human embryonic kidney cell line HEK293T (CRL-3216, ATCC, VA, USA) was cultured in DMEM (Cat. 12100-046; Lot. 1651558, Thermo Fisher, MA, USA) with 10% FBS and 1% L-glutamine. Cells were cultured in a 5% CO_2_, 37 °C humidified incubator and medium was refreshed every 2 days.

### Construction of wild-type and mutant HAI-2 expression vector

We used the mammalian expression vector pcDNA3.1/myc-His (V80020, Thermo Fisher, MA, USA) to express wild-type and mutant HAI-2 proteins in prostate cancer cells. The DNA fragment encoding HAI-2 proteins was amplified using PCR from the cDNA pool of MDA-MB-231 cells, and ligated into pcDNA3.1/myc-His using *HindIII* and *XhoI* restriction sites. The c-Myc and His tags were fused with the carboxyl terminus of HAI-2 in the construction. The mutants of HAI-2 were then created by site-directed mutagenesis (Supplementary Table S1) using HAI-2.pcDNA3.1/myc-His as a template.

### Transfection and establishment of stable HAI-2-expressing N2 cells

Cells were seeded at a density of 3 × 10^6^ cells per 60-mm dish. Next day the medium was refreshed with 3 ml of OPTI-MEM (Cat. 31985070, Thermo Fisher, MA, USA). For transfection, 0.5 ml of transfection solution [a mixture of 4 µg plasmid DNA and 6 µl Lipofectamine 2000 (Cat. 11668019, Thermo Fisher, MA, USA)] were added into the culture medium. After 6-hour incubation, the medium was discarded and refreshed with the regular culture medium and then the cells were cultured for 2 days. For the establishment of stable pools, transfected cells were selected with 750 µg/ml G418 (Cat. 10131027, Thermo Fisher, MA, USA) under a regular culture condition for two weeks.

### Genetic knockdown by shRNA with lentiviral particles

The plasmids of shRNA clones against HAI-2 (#1 TRC0000073578 and #2 TRC0000073579) and a lentiviral production system were purchased from the RNAi Core Facility, Academia Sinica, Taipei, Taiwan. The methods for viral particle production and infection were referred to the protocols provided by the RNAi Core Facility. Briefly, the plasmids for shRNA lentiviral particle packaging were co-transfected to HEK293T cells. After 48 hours, the conditioned media were collected as lentiviral particle solutions. To infect cells, 8 µg/ml of polybrene (Hexadimethrine bromide, Cat. H9268, Lot. 028K2581, Sigma-Aldrich, MO, USA), 1 ml of virus solution and 2 ml of regular medium were mixed and added to the cells. Next day, the media were refreshed. The infected cells were recovered for 24 hours, and then selected with 2 µg/ml puromycin (Cat. ant-pr, Lot. QLL-38-01A, Sigma-Aldrich, MO, USA) for 2 weeks under regular culture conditions.

### Western blot

Cells were lysed with lysis buffer [1% Triton X-100 (Cat. X198-05, Lot. 003623, J.T.Baker, PA, USA) in PBS (Phosphate Buffered Saline, Cat. 099400-100, Lot. 222708, pH 7.4, Medicago, Canada) supplied with a protease inhibitor cocktail (Cat. 14975900, Lot. Sep 2010, Roche, Switzerland)] and then centrifuged at 13,000 r.p.m., 4 °C for 15 min. The lysate was collected and their protein concentrations were measured by Bradford assay (following manufacture’s protocol, Cat. 500-0006, Lot. 64038229, Bio-Rad, CA, USA). Equal amounts of each protein sample was mixed with SDS sample buffer [8% SDS (sodium dodecyl sulfate, Cat. 20765, Lot. 150511, SERVA, German), 40% glycerol (Cat. G7893, Lot. SHBC3297V, Sigma-Aldrich, MO, USA), 0.008% bromophenol blue (Cat. 114391, Lot. 53180, Sigma-Aldrich, MO, USA) and 0.25 M Tris pH 6.8] plus 5% β-mercaptoethanol (Cat. M3148, Lot. BCBF9538V, Sigma-Aldrich, MO, USA)] and boiled at 95 °C for 10 min. Samples were then used for SDS-PAGE and immunoblot analysis.

The M32 and M69 mAbs were gifts from Dr. Chen-Yong Lin at the Georgetown University, Washington, DC, USA, and generated as briefly described as follows: For the monoclonal antibody preparation, BALB/c mice were immunized by the matriptase-HAI-1 complexes, which were purified from human milk^71^. The splenocytes of the mice were then isolated and fused with myeloma cells to generate hybridoma. The hybridoma producing Abs were screened by western blots using purified matriptase complexes. Two clones of hybridoma were selected and could produce specific Abs that could recognize the third LDLR domain (M32) and activated protease domain (M69) of matriptase^71^. These mAbs were first isolated by precipitation using 50% saturation of ammonium sulfate. Further purification was performed by anion exchanger (DEAE) chromatography. The samples for the examination using these two Abs were prepared under non-boiling and non-reducing conditions. Equal amounts of samples were loaded for SDS-PAGE and then transferred to nitrocellulose membrane (Cat. 10600002; Lot. 9917808, GE Life Science, CT, USA). The membranes were blocked with 5% skim milk in TBST buffers [Tris-buffered saline: 50 mM Tris (Tris-base Cat. 4109-01; Lot. L06600, J.T.Baker, PA, USA), 150 mM NaCl (Cat. 31434, Lot. SZBF1590V, Sigma-Aldrich, MO, USA), plus 0.1% Tween-20 (Cat. X251-07, Lot. 0000140203, J.T.Baker, PA, USA)], and then incubated with diluted primary antibodies overnight [anti-HAI-2 rabbit pAb (customized by Kelowna, Taipei, Taiwan), anti-c-Myc mouse mAb (9E10, Cat. sc-40, lot. I1415, Santa Cruz, CA, USA), anti-matriptase rabbit pAb (Cat. IM1014, Lot. 2733415, Millipore, CA, USA), anti-GST mAb (Cat. YH80001, Yao-Hong Bio, Taipei, Taiwan), anti-His mAb (Cat. MA1-21315, Lot. RA225460, Thermo Fisher, MA, USA), and anti-β-actin mAb (AC-15, Cat. A5441, lot. 061M4808, Sigma-Aldrich, MO, USA)]. After three washes of TBST, the membrane was incubated with a secondary antibody conjugated with HRP (Horseradish peroxidase) [HRP-conjugated goat anti-rabbit IgG (Cat. 111-035-144, Lot. 95092, Jackson immunoresearch, PA, USA) and HRP-conjugated goat anti-mouse IgG (Cat. 115-035-003, Lot. 103787, Jackson immunoresearch, PA, USA] for 1 hr. To visualize the images, ECL (Enhanced ChemiLuminescent) reagent (Western Lightning Plus-ECL, Cat. 0RT2655, Lot. 265-15351, PerkinElmer, MA, USA) was applied and the signals were captured by a CCD camera (LAS-4000, Fujifilm, Japan).

### Transwell assay

The cell motilities (including cell migration and invasion) were determined by Boyden chamber assay that was described previously^96^. For cell invasion assay, 2 µl of Matrigel (Cat. 354234, Lot. 5215010, Corning, NY, USA) were mixed with 100 μl of ddH_2_O, evenly coated on a Boyden chamber (Millicell Hanging insert, Cat. PIE12R48, Lot. 14220116, Millipore, CA, USA) and air-dried overnight. The Matrigel was reconstituted with serum-free medium before usage. For cell migration assay, no Matrigel was coated. After starvation with serum-free medium for 16 hours, cells were seeded into Boyden chambers with 200 µl serum-free medium per chamber. The chambers were then placed in a well of 24-well plates which was filled with 700 µl of regular medium per well. After the incubation for the indicated times, cells were fixed with 100% methanol for 10 min and then stained with 1% crystal violet (Cat. C3886, Lot. 086K0052, Sigma-Aldrich, MO, USA) for 10 min. The cells on the upper chambers of transwells were removed by cotton swabs. The migrated and invaded cells on the bottom chambers of transwells were photographed by a microscopy, and analyzed with ImageJ software v.1.50i (National Institutes of Health, MD, USA) for statistical calculations from three independent experiments.

### Immunofluorescence and confocal microscopy

Cells were seeded on glass cover slips and then fixed with 4% paraformaldehyde (Cat. 1.04005.1000, Lot. K44440405, Merck Millipore, German) in PBS at 80 °C for 30 min. After two washes by 0.1 M of glycine (Cat. 4059-06, Lot. 0000130732, J.T.Baker, PA, USA), cells were blocked with 5% goat normal serum (Cat. 31873, Thermo Fisher, MA, USA) plus 1% BSA (Bovine serum albumin, Cat. UR-BSA-001-100G, UniRegion Biotech, Taipei, Taiwan) in PBS. Samples were then incubated with primary antibodies in 1% BSA PBS at 4 °C overnight. After washing with 0.1% Triton X-100 PBS, secondary antibodies conjugated with rhodamine (goat anti-rabbit IgG cross-absorbed secondary antibody, rhodamine, Cat. 31686, Thermo Fisher, MA, USA) or FITC (Fluorescein isothiocyanate, goat anti-mouse IgG cross-adsorbed secondary antibody, FITC, Cat. 31569, Thermo Fisher, MA, USA) were added and incubated with the samples for 1 hour. Hoechst 33342 solution (Excitation/Emission (nm): 361/497, Cat. H3570, Thermo Fisher, MA, USA) was employed to visualize nuclei. Samples were mounted with ProLong Gold antifade reagent (Cat. P36930, Lot. 1618291, Thermo Fisher, MA, USA) and then fluorescent images were captured by a confocal microscopy (TCS SP5, Leica, German).

### Immunoprecipitation

Cells were lysed with immunoprecipitation buffer [0.5% NP-40 (IGEPAL® CA-630, Cat. I8896, Lot. MKBN1103V, Sigma-Aldrich, MO, USA), 150 mM NaCl, and 50 mM Tris pH 7.0], and the equal amounts of lysates were collected and incubated with primary antibodies [anti-c-Myc mouse mAb (9E10, Cat. sc-40, lot. I1415, Santa Cruz, CA, USA); anti-V5 mouse mAb (ab27671, Lot. GR186433-4, Abcam, UK)] at 4 °C overnight. Pre-washed protein G magnetic beads (Protein G Mag Sepharose Xtra, Cat. 28-9670-70, Lot. 10232014, GE Life Science, CT, USA) were then added and incubated for 1 hour. The beads were precipitated and washed three times with PBS. To elude the target proteins, the beads were mixed with SDS sample buffer followed by boiling.

### Purification of recombinant matriptase protease domain (Trx-MTX PD)

The DNA fragment containing the nucleotide sequence of matriptase protease domain (MTX PD, amino acid sequence from 615 to 854) was amplified using PCR and cloned to pET32a vector (Millipore, CA, USA) containing a thioredoxin reductase tag (Trx). Trx-MTX PD plasmid was then used for transformation of BL21 (DE3) *E. coli*. For the protein expression, the *E. coli* cells were treated with 0.5 mM of IPTG (Isopropyl β-D-1-thiogalactopyranoside, Cat. UR-IPTG-25G, UniRegion Bio-tech, Taipei, Taiwan) for 4 hours. Since Trx-MTX PD polypeptides were mainly found in the inclusion bodies of the cell lysates, to obtain the functional protease activity, the pellets were then dissolved in refolding buffer [8 M urea (Cat. 4204-05, J.T.Baker, PA, USA), 10 mM DTT (dithiothreitol, Cat. D9779, Lot. SLBK2997V, Sigma-Aldrich, MO, USA) in PBS, pH 8.0]. The Trx-MTX PD polypeptides were then purified by Talon resin (Cat. 28-9574-99, Lot, 10226094, GE Life Science, CT, USA) following manufacture’s protocol. For protein refolding, the concentration of the purified Trx-MTX PD polypeptides was diluted to 0.5 mg/ml in refolding buffer. Ten milliliters of the protein solution (5 mg of protein samples) were taken and loaded into a cellulose dialysis tube (Orange Scientific, Belgium). The dialysis tube was immerged into 1 L of PBS (pH 7.4) containing 4 M urea with stirring at 4 °C overnight. Next day, the dialysis tube was moved to 1 L PBS containing 2 M urea with stirring at 4 °C overnight. Following the same dialysis procedure, the concentration of urea was daily half reduced in PBS for the protein refolding (a stepwise dialysis with the serial decreases of urea concentrations from 4 M, 2 M, 1 M, to 0.5 M). To collect the functional protease domain proteins, the protein solution was dialyzed with 1 L PBS twice to remove any trace of urea, and then centrifuged (13,000xg, 15 min) to precipitate pellets. The supernatant was then collected and stored at −80 °C for the experiments of proteolytic activity assays.

### Purification of GST fusion proteins

The DNA fragments encoding HAI-2’s KD1 and KD2 were amplified using PCR and cloned to pGEX 4T-1 vectors with a GST tag (GE Life Science, CT, USA). To generate GST-KD1 and GST-KD2 proteins, BL21 (DE3) *E. coli* cells were transformed by the KD1- or KD2-pGEX 4T-1 plasmids, and then received 0.5 mM IPTG induction for 4 hours at room temperature. The cells were collected and lysed by sonication (20% amplitude with 5 sec on and 10 sec off in 120 cycles, on ice) in pH 7.4 PBS containing 1% Triton X-100 (Cat. X198-05, Lot. 003623, J.T.Baker, PA, USA) and 1 mM phenylmethylsulfonyl fluoride (Cat. P7626, Sigma-Aldrich, MO, USA)]. Since GST-KD1 fusion proteins were majorly found in unsolved fraction, to prepare the functional GST-KD1 proteins, the fusion proteins were dissolved in 8 M urea solution and then underwent the refolding process (following the same procedure as mentioned above) before affinity purification. For protein purification, 10 ml of 0.5 mg/ml refolded products (GST-KD1) or cell lysate (GST and GST-KD2) were passed through an affinity column with 0.5 ml glutathione sepharose (Glutathione Sepharose 4B, Cat. 17-0756-01, Lot. 10049253, GE Life Science, CT, USA) by gravity. The sepharose beads were washed twice with 3 ml of wash buffer (20 mM phosphate buffer, pH7.4, 450 mM NaCl). The proteins were eluted by 3 ml of elution buffer [20 mM of reduced glutathione (Cat. G4251, Lot. 030M1775V, Sigma-Aldrich, MO, USA) in 50 mM Tris-base, pH 8.0].

### Matriptase activity assay

Purified matriptase protease domain (Trx-MTX-PD) proteins were mixed with the indicated concentrations of purified recombinant HAI-2 proteins in PBS (pH 7.4) at a final volume of 50 μl, and incubated for 30 min at room temperature. The artificial substrate, Boc-Gln-Ala-Arg-AMC (7-Amino-4-methylcoumarin, Cat. BML-P237-0005, Lot. 04071614, Enzo life science, NY, USA), was used to measure the *in vitro* proteolytic activity of the serine protease^97^. Briefly, the active protease can cleave the substrate to release the AMC groups, which can be excited by UV light and emits blue light (EX: 360 nm; EM: 465 nm). For the protease reaction, 50 μl of the substrate (100 µM) were mixed with 50 μl of the enzyme-inhibitor solution in a well of 96-well black plate (Cat. 237108, Nunc MicroWell black polystyrene plate, Thermo Fisher, MA, USA) and kept at 37 °C for the indicated times. The intensity of fluorescence was detected by a microplate reader (SpectraMax Paradigm, Beckman Coulter, CA, USA) at each time point, and statistically calculated as an equation of [ΔRFU (relative fluorescent unit) = RFU_end_ − RFU_0_] to represent the protease activities.

### Baculovirus expression system and recombinant HAI-2 (rHAI-2) protein purification

The DNA fragments for encoding the extracellular region and mutants of HAI-2 were synthesized by GeneArt (Thermo Fisher, MA, USA), and cloned to pFastBac Dual vector (Thermo Fisher, MA, USA) which contains a melittin signal peptide in the amino terminus and a 6xHis tag in the carboxyl terminus. The plasmids were then used to transform DH10Bac *E. coli* for genetic recombination to generate recombinant bacmids (Bac-to-Bac system, Thermo Fisher, MA, USA). To produce recombinant baculoviral particles, 2.5 µg of bacmids were used to transfect 1 × 10^6^ of High Five cells (Cat. B85502, Thermo Fisher, MA, USA) using transfection reagent (TransIT-Insect, Cat. MIR 6104, Mirus Bio, WI, USA) following manufacture’s protocol. The viral particles were then used to infect High Five cells for the production of rHAI-2 by the titerless method^98^, as briefly described as follows. High Five cells were infected with recombinant baculoviral particles to generate baculovirus-infected insect cells, abbreviated as BIICs. BIICs were then co-cultured with un-infected High Five insect cells at a ratio of 1:100 for 3~5 days until the cell growth stops. Three hundred milliliters of the conditioned media balanced at pH 7.2 were fractionated by a cation exchanger chromatography (5-ml cation exchanger column, Cat. 17115201, Lot. 10233976, Hitrap SP HP, GE, Life Science, CT, USA). The sample was then washed with 25 ml of 20 mM phosphate buffer (pH 7.2) containing 100 mM NaCl. The proteins were eluted by 30 ml of 20 mM phosphate buffer (pH 7.2) containing 200 mM NaCl. The eluted proteins further underwent through an affinity purification of a Talon resin column (Cat. 28-9574-99, Lot, 10226094, GE life science, CT, USA) following manufacture’s protocol.

### SPR-based BIAcore assay

The series S sensor chip CM5 (Cat. 29104988, Lot. 10071039, GE Life Science, CT, USA) was activated using the solution [0.05 M of NHS (N-Hydroxysuccinimide) and 0.2 M of EDC (N-ethyl-N’-dimethylaminopropyl carbodiimide) (Amine couple kit, Cat. BR-1000-50, GE Life Science, CT, USA)] at a flow rate of 5 µl/min for 7 min. To immobilize matriptase protease domain (Trx-MTX PD) proteins in the S sensor chip CM5, 0.2 μg/ml of Trx-MTX PD proteins in 10 mM sodium acetate (pH 5.0) buffer (Cat. BR100351, GE Lifescience, CT, USA.) were passed through the chip at a flow rate of 5 µl/min for 10 min. The remaining coupling reagent in the chip was then quenched with 1 M ethanolamine, which was provided in the amine couple kit (Cat. BR-1000-50, GE Life Science, CT, USA). The analytes (GST, GST-KD1 and GST-KD2 fusion proteins) were diluted and dialyzed in a running buffer [10 mM HEPES (4-(2-hydroxyethyl)-1-piperazineethanesulfonic acid, Cat. H3375, Sigma-Aldrich, MO, USA), 150 mM NaCl, pH 7.4], and then injected through the chip at a flow rate of 30 µl/min for 120 sec. To dissociate the analytes, the chip was washed with the running buffer at a flow rate of 30 µl/min for 180 sec. Between each cycle, the chip was regenerated with 10 mM of glycine-HCl solution, pH 2.5 (Regeneration scouting kit, Cat. BR100556, GE Life Science, CT, USA) at a flow rate of 30 µl/min for 30 sec. The dissociation constants (K_D_) were statistically calculated by affinity analysis of BIAevaluation software v1.0 (GE Life Science, CT, USA).

### Computation of protein-protein docking

The structural models of matriptase protease domain (4ISO), HAI-1’s KD1 (4ISO), HAI-2’s KD1 (4U32) were downloaded from the Protein Data Bank (http://www.rcsb.org/pdb/home/home.do). The model of HAI-2’s KD2 was constructed by SWISS-MODEL (https://swissmodel.expasy.org/)^75,76^ using HAI-2’s KD1 model (4U32) as a template. The docking models between HAI-2’s KD1 or KD2 with matriptase protease domain (4ISO) were performed by ClusPro 2.0^77–81^. The residues in the reactive region of each polypeptide^40^ (amino acid residue 189–190 in the matriptase protease domain, amino acid residue 48–50 in HAI-2’s KD1, and amino acid residue 143–145 in HAI-2’s KD2) were set as attraction residues in the advanced options. The models in cluster 0 (that has the highest number of members) using “balanced” coefficients were chosen. The docking models were drawn by CCP4mg software v.2.10.6 (MRC Laboratory of Molecular Biology, Cambridge, UK).

### Statistical analysis

A mean ± S.D. was statistically calculated form three independent experiments. Statistical significance between two groups was determined by One-way ANOVA using Prism software (GraphPad, CA, USA).

## Electronic supplementary material

Supplementary information
