# Author Correction: The Kunitz Domain I of Hepatocyte Growth Factor Activator Inhibitor-2 Inhibits Matriptase Activity and Invasive Ability of Human Prostate Cancer Cells

**DOI:** 10.1038/s41598-020-60001-w

**Published:** 2020-02-13

**Authors:** Shang-Ru Wu, Chen-Hsin Teng, Ya-Ting Tu, Chun-Jung Ko, Tai-Shan Cheng, Shao-Wei Lan, Hsin-Ying Lin, Hsin-Hsien Lin, Hsin-Fang Tu, Pei-Wen Hsiao, Hsiang-Po Huang, Chung-Hsin Chen, Ming-Shyue Lee

**Affiliations:** 1 Department of Biochemistry and Molecular Biology, College of Medicine, National Taiwan University, Taipei, Taiwan; 2 Agricultural Biotechnology Research Center, Academia Sinica, Taipei, Taiwan; 3 Graduate Institute of Medical Genomics and Proteomics, College of Medicine, National Taiwan University, Taipei, Taiwan; 4 Department of Urology, National Taiwan University Hospital, Taipei, Taiwan

Correction to: *Scientific Reports* 10.1038/s41598-017-15415-4, published online 08 November 2017

In Figure 1D, the column ‘103E’ is incorrectly given as ‘N1’. In Figure 5A, the kDa for the blot GST-HAI-2 KD ‘43’ is incorrectly given as ‘55’. The correct Figure 1 and Figure 5 appear below as Figure 1 and Figure 2 respectively.

**Figure 1:**
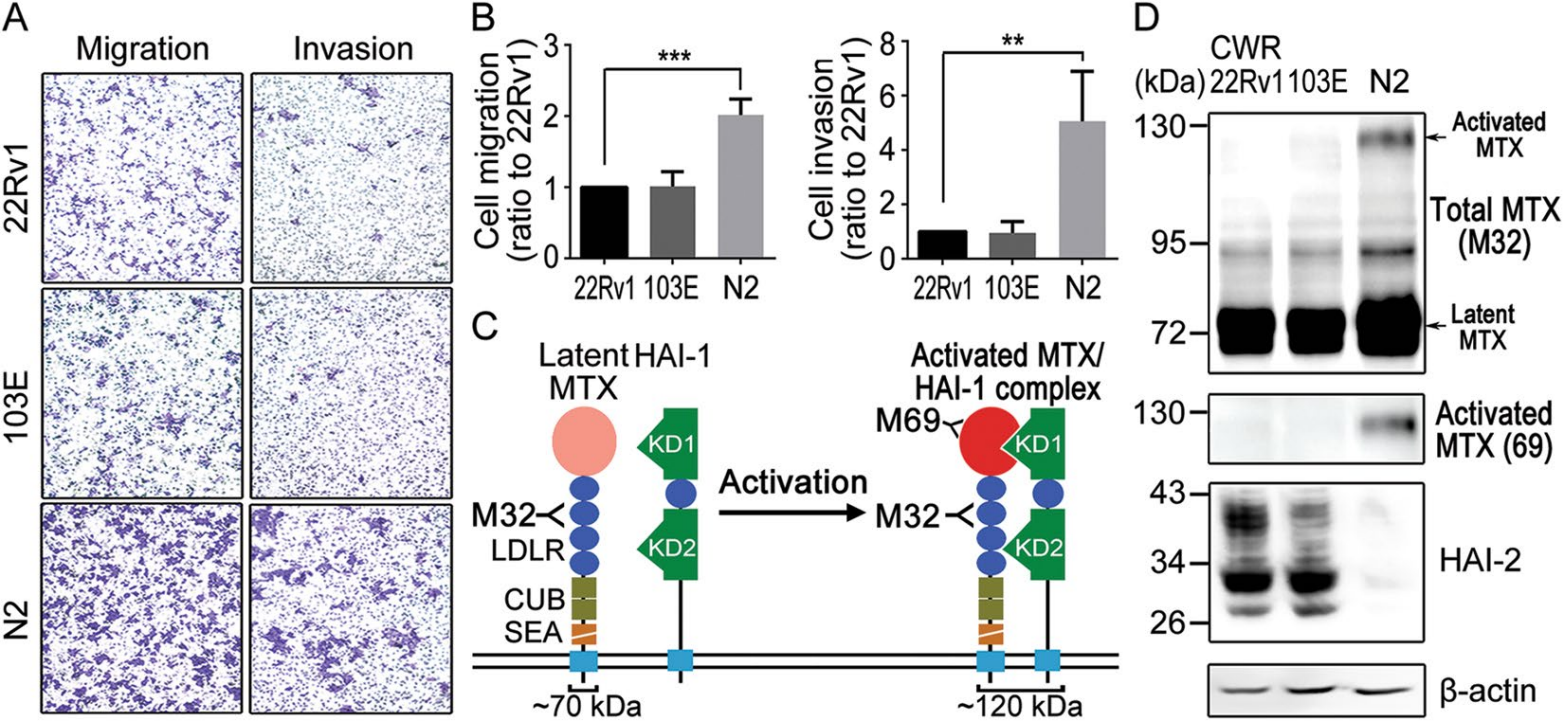
.

**Figure 2:**
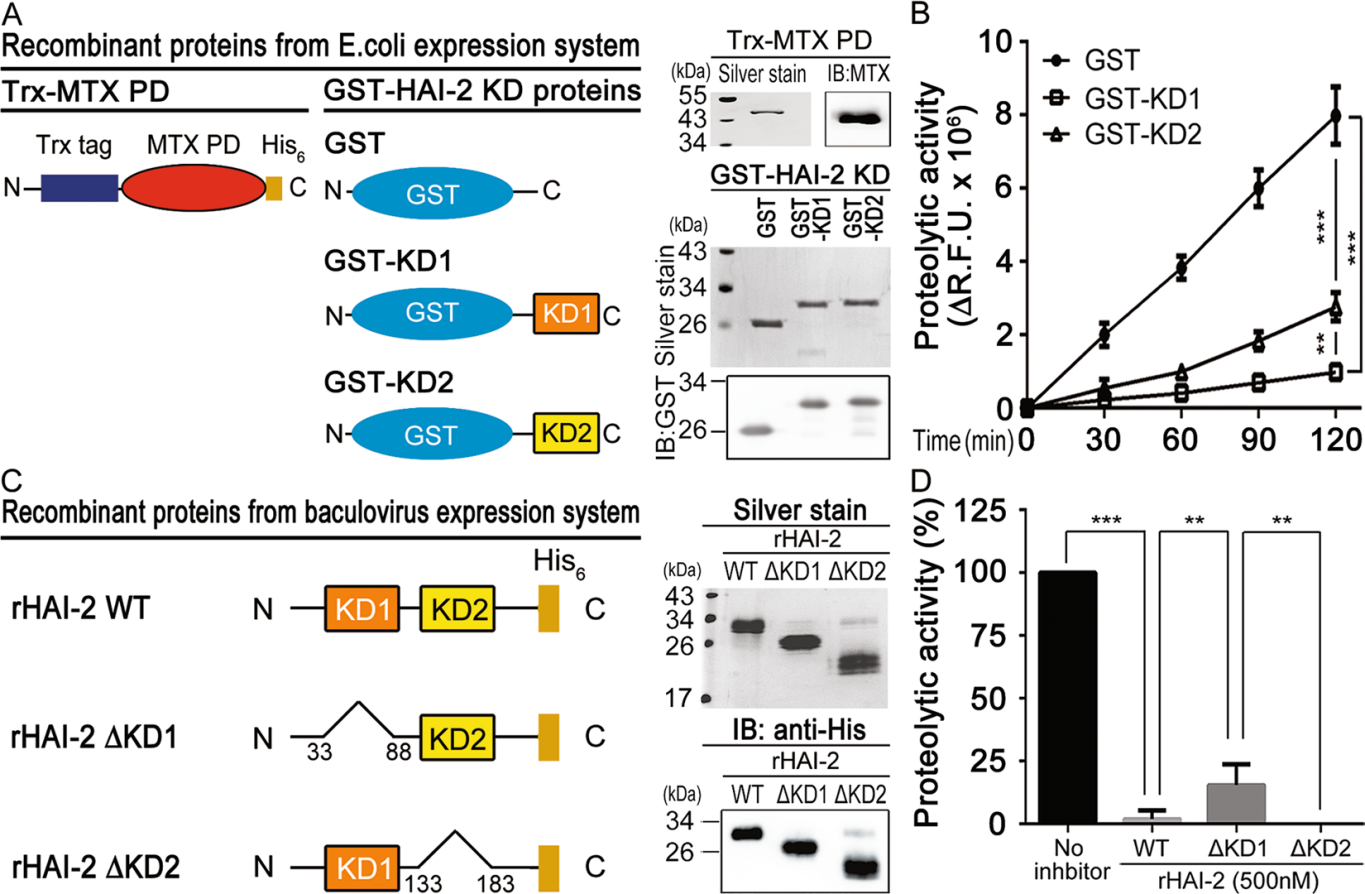
.

