# Supplementary material for: The Kunitz Domain I of Hepatocyte Growth Factor Activator Inhibitor-2 Inhibits Matriptase Activity and Invasive Ability of Human Prostate Cancer Cells
Source: Sci Rep. 2017 Nov 8;7:15101. doi: 10.1038/s41598-017-15415-4 (PMC5678078; doi:10.1038/s41598-017-15415-4)
Supplement: Supplementary file 1 — Supplementary information [file 41598_2017_15415_MOESM1_ESM.pdf]

Supporting Information

The Kunitz Domain I of Hepatocyte Growth Factor Activator Inhibitor-2 Inhibits Matriptase Activity and Invasive Ability of Human Prostate Cancer Cells

Shang-Ru Wu[1], Chen-Hsin Teng[1], Ya-Ting Tu[1], Chun-Jung Ko[1], Tai-Shan Cheng[1], Shao-Wei Lan[1], Hsin-Ying Lin[1], Hsin-Hsien Lin[1], Hsin-Fang Tu[1], Pei-Wen Hsiao[2], Hsiang-Po Huang[3], Chung-Hsin Chen[4] and Ming-Shyue Lee[1*]

[1]Department of Biochemistry and Molecular Biology, College of Medicine, National Taiwan University; [2]Agricultural Biotechnology Research Center, Academia Sinica; [3]Graduate Institute of Medical Genomics and Proteomics, College of Medicine, National Taiwan University; [4]Department of Urology, National Taiwan University Hospital, Taipei, Taiwan.

[*]Corresponding author: Ming-Shyue Lee, Ph.D.

Department of Biochemistry and Molecular Biology, College of Medicine, National Taiwan University. R817, 8F, No. 1, Section 1, Jen-Ai Rd. Taipei, Taiwan

Tel. 011-886-2-2395-7966, FAX: 011-886-2-2391-5295

E-mail: mslee2006@ntu.edu.tw

Running title: KD I of HAI-2 in matriptase and prostate cancer

Conflict of Interest: No potential conflicts of interest were disclosed.

Legends of Supplementary Figures

Figure S1. The growth of HAI-2-silencing CWR22Rv1 cells. CWR22Rv1 cells were infected with lentiviral particles with HAI-2 shRNAs (#1 and #2) and selected by puromycin (2 μg/ml) for two weeks. Cells were seeded at a density of $2.5x10^3$ cells per well of 96-well dishes. One day after seeding, cell growth was examined as Day 0 using MTT assays, according to the protocol (Sigma-Aldrich, MO). The cell growth on Day 1, 2 and 3 were further analyzed and normalized to Day 0. Data were statistically calculated and presented as mean±S.D. from three independent experiments.

Figure S2. Examination of HAI-2-knockdown CWR22Rv1 and matriptase- or HAI-2-overexpressing 103E cell migration and invasion. (A) Analyses of HAI-2-silencing CWR22Rv1 cell migration and invasion using cell motility assays. Cells were seeded at a density of $2x10^5$ cells in an upper well of Boyden chambers for cell migration (24 hours) and invasion assays (48 hours). The migrating and invasive cells on the bottoms of Boyden chamber were photographed. The images from the transwell assays were taken with a magnification of x100 and a set of representatives was shown from three independent experiments. (B) Examination of matriptase- and/or HAI-2-overexpressing 103E cell migration and invasion. 103E cells were stably transfected with the plasmids to express matriptase (MTX) and/or HAI-2. The cell migration and

invasion were assessed by transwell assays. Cells were seeded at a density of $3x10^5$ cells in an upper well of Boyden chambers and incubated for 20 hours. The invading and migrating cells on the bottom of the transwells were photographed. The images of 103E cell migration and invasion were visualized with a magnification of x100 and a set of representatives was shown from three independent experiments.

Figure S3. The recombinant matriptase protease domain with thioredoxin tag (Trx-MTX PD) was purified from an *E.coli* expression system by Talon resin (GE, MA) under denatured condition and refolded into soluble forms (for details, see Materials and Methods). One μg of Trx-MTX PD was loaded into SDS-PAGE and revealed by silver staining.

Figure S4. Analysis of the effects of purified rHAI-2 proteins on N2 and PC-3 cell migration and invasion. Cells were seeded at a density of $2x10^5$ (N2) or $5x10^4$ (PC-3) cells per well of Boyden chambers and treated with the indicated concentrations of rHAI-2 proteins. Boyden chambers were coated with or without Matrigel before cell seeding for cell invasion and migration assays. Migration assays were performed for 24 hours (N2) and 16 hours (PC-3) and invasion assays were carried out for 48 hours (N2) and 24 hours (PC-3) in the presence of the indicated concentrations of rHAI-2 proteins

(R&D Systems, Inc., MN). The invading and migrating cells on the bottoms of transwells were photographed with a magnification of x100. The images were a set of representatives from three independent experiments.

Figure S5. Analyses of purified recombinant HAI-2 proteins using silver staining and immunoblotting. (A) GST (glutathione-S-transferase) and GST-fusion proteins (GST, GST-KD1 and GST-KD2) were purified from an *E.coli* expression system by glutathione-beads (GE, MA) (for details, see Materials and Methods). 0.5 μg of GST-fusion proteins were separated by SDS-PAGE and revealed by silver stain as well as (B) immunoblotting with anti-GST. The purities of the proteins were quantified by ImageJ v1.50i (NIH, MD, USA). (C) Recombinant HAI-2 (rHAI-2) proteins were purified from a baculovirus expression system using an affinity column (for details, see Materials and Methods). The purified proteins (0.5 μg per sample) were subjected to SDS-PAGE for silver stain and (D) immunoblotted using an anti-His antibody (Cat. MA1-21315, Thermo Fisher, MA, USA).

Figure S6. Examination of the effects of HAI-2 mutants on cell migration and invasion. N2 cells stably expressing HAI-2 mutants were seeded at a density of $3x10^5$ cells in an upper well of Boyden chamber and incubated for 16 hours. The invading and migrating

cells on the bottom of each transwells were photographed. The images were taken with a magnification of x100 and a set of representatives from three independent experiments were shown.

Figure S7. Examination of the effects of purified recombinant HAI-2 variant proteins on N2 and PC-3 cell migration and invasion. Cells were seeded at a density of $2x10^5$ (N2) and $5x10^4$ (PC-3) cells per well of Boyden chambers and treated with 50 nM of purified wild-type (WT) and domain-deleted mutants (ΔKD1 and ΔKD2) of rHAI-2 proteins (from baculovirus expression system). Migration assays were performed for 24 hours (N2) and 16 hours (PC-3), and invasion assays were carried out for 48 hours (N2) and 24 hours (PC-3). The migrating and invading cells on the bottoms of transwells were photographed with a magnification of x100. The images were a set of representatives from three independent experiments.

Figure S8. HAI-2 complexes with matriptase and reduces the matriptase-HAI-1 complex in the presence of HAI-1. (A) HEK293T cells were seeded at 60% confluence and transfected with plasmids of matriptase (MTX) or/and HAI-2. Two days after transfection the lysates were collected for Western blot. The matriptase complexes were detected by M69 and M32 mAbs under a non-boiled and non-reducing condition. (B)

Different amounts of plasmids of matriptase, HAI-1 and HAI-2 were transfected to HEK293T cells. Two days after transfection, the lysates were collected for Western blot. The matriptase complexes were detected by M69 and M32 mAbs under a non-boiled and non-reducing condition. The matriptase-HAI-1 complex was detected by M19 mAb under a non-boiled and non-reducing condition. (-, only vector for transfection. +, one-fold amount of plasmid for transfection. ++, two-fold amount of plasmid for transfection.)

Figure S9. The N-glycan depletion of endogenous HAI-2, exogenous HAI-2 and recombinant HAI-2. (A) The prostate cancer cell line DU145 $3x10^6$ cells were seeded at 10-cm dishes and grew for 24 hr in the absence/presence of 1 μg/ml tunicamycin (Cat. T7765, Lot. 081M4075V, Sigma-Aldrich, MO, USA). The 40 μg of lysate samples were analyzed by immunoblotting with anti-HAI-2 pAb. (B) $5x10^5$ 293T cells were seeded at a well of 6-well plate. Next day 0.5 μg plasmid mixed with 1 μl lipofectamine 2000 (Cat. 11668019, Thermo Fisher, MA, USA) were added to cells. Next day 1 μg/ml Tunicamycin (Cat. T7765, Sigma-Aldrich, MO, USA) was added to the cells (+) or not (-). After 24 hr, the cells were collected and 20 μg of cell lysate was subjected to immunoblotting using anti-c-Myc mAb (9E10, Cat. sc-40, Santa Cruz, TX, USA). (C) 100 ng of recombinant HAI-2 purchased from R&D system (Cat. 1106-PI-010,

produced from the mouse NS0 cell line) were treated with PNGase F (Cat. P0704S, NEB, MA, USA) following manufacture's protocol. Briefly, recombinant HAI-2 was incubated in Denaturing buffer (provided in the kit) and heated to 99°C for 10 min. One μl of PNGase F as well as 1% NP-40 were then mixed with the sample and incubated for an hour. The proteins with/without the treatment were analyzed by immunoblotting using anti-HAI-2 pAb.

Figure S10. Subcellular location of HAI-2 is revealed by immunofluorescence and a confocal microscopy. N2 prostate cancer cells were seeded on a cover slip and transiently expressing HAI-2 tagged with c-Myc tag. After fixation and permeabilization, the sample was covered by anti-c-Myc pAb (to recognize HAI-2, sc-788, Santa Cruz) and anti-E-cadherin mAb (Cat. 610181, BD bioscience) overnight. After three washes with 0.1% Triton X-100 PBS, rhodamine-conjugated anti-rabbit Ab (Red) and FITC-conjugated anti-mouse Ab (Green) as secondary Abs were used to detect the HAI-2 and E-cadherin (E-cad), respectively. The arrow indicates the co-localization of HAI-2 and E-cadherin around cell surface. The image was captured by a confocal microscopy (TCS SP5, Leica).

Figure S11. Cell-surface biotinylation and pulldown reveal the presence of HAI-2 on

the cell surface of N2 prostate cancer cells. $3x10^6$ N2 cells with or without HAI-2 expression were seeded in 10-cm dishes and incubated for 3 days. After two washed with ice-cold PBS pH 8.0, the cells were covered with 80 μM sulfo-NHS-SS-biotin (Cat. 21331, Thermo Fisher) in PBS pH 8.0 at 4°C for an hour. The cells were then lyzed and the lysate was incubated with streptavidin beads (Streptavidin Mag Sepharose, Cat. 28-9857-38, GE Life Sciences) at 4°C for 30 min. After four washes with lysis buffer, the captured biotinylated proteins were eluted by 2-mercaptoethanol and SDS sample buffer in a boiling condition. Whole lysate is the input sample. The flow through is labeled as an intracellular fraction, and the biotinylated fraction is labeled as a pericellular fraction. E-cadherin (E-cad) and β-actin were used as control.

Figure S12-S18. The original data of immunoblotting displayed in the main figures. Red rectangles indicate the cropped regions.

Table S1. The primer used to generate HAI-2 mutations. The mutants of HAI-2 were created by site-directed mutagenesis. Briefly, 50 ng of HAI-2 plasmid (HAI-2. pCDNA 3.1-Myc/His) as template was mixed with 2.5 U pfuTurbo DNA polymerase (Cat. 600250, Agilent, CA, USA), 3 mM of each forward/reverse primer and 10mM dNTP in 50 μl reaction buffer. The PCR was carried out by Thermocycler PC320 (Astec, Japan)

under the condition as follows; 17 thermo-cycles with 3 steps: (1) denaturing at 95°C for 30 sec. (2) annealing at 60 °C for 1 min. (3) extension at 72°C for 14 min. The products were treated with 20 U DpnI (Cat. R0176S, NEB BioLabs, MA, USA) to remove the templet and then transformed to *E.coli*.

Figure S1

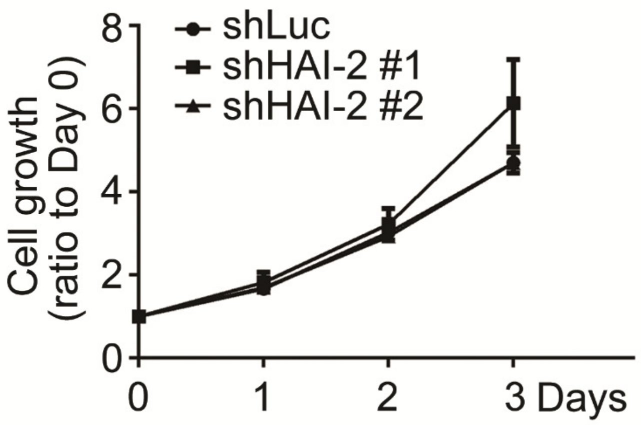
shLuc
shHAI-2 #1
shHAI-2 #2
Cell growth
(ratio to Day 0)
8
6
4
2
0
0
1
2
3 Days

Figure S2

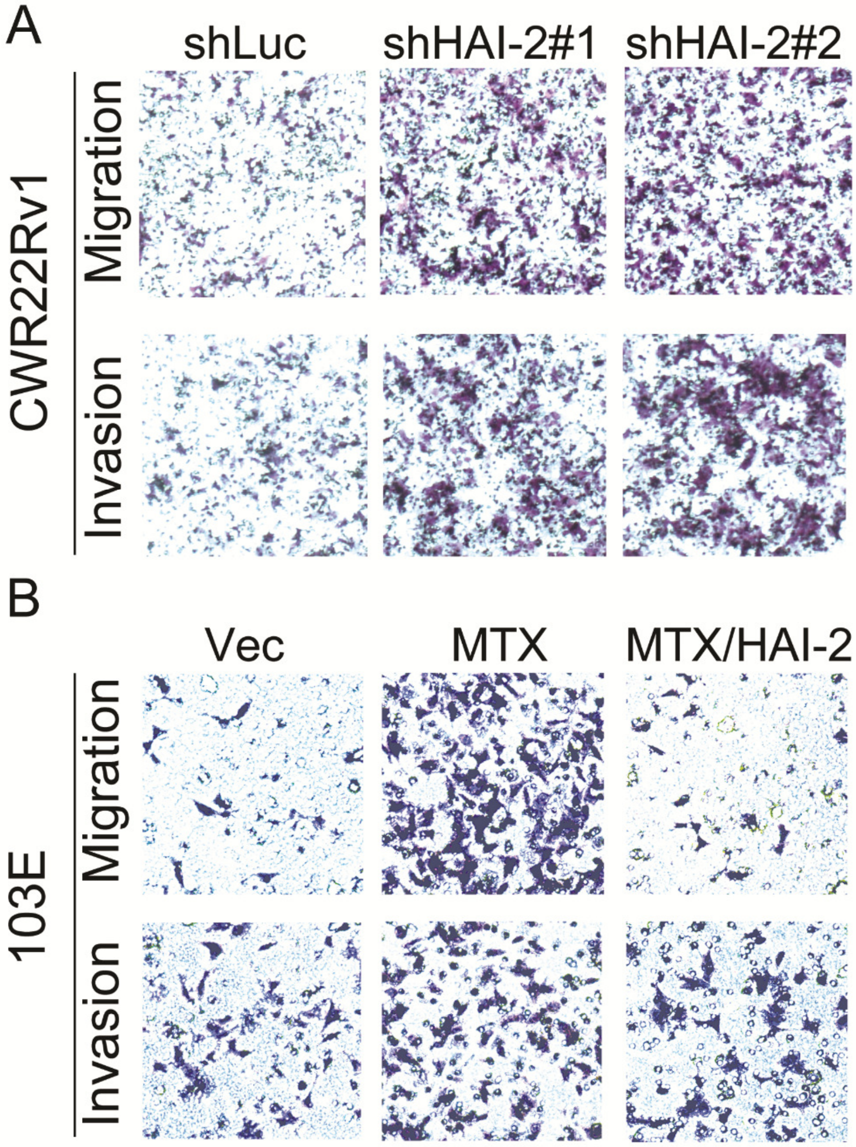
A
shLuc
shHAI-2#1
shHAI-2#2
CWR22Rv1
Migration
Invasion
B
Vec
MTX
MTX/HAI-2
103E
Migration
Invasion

Figure S3

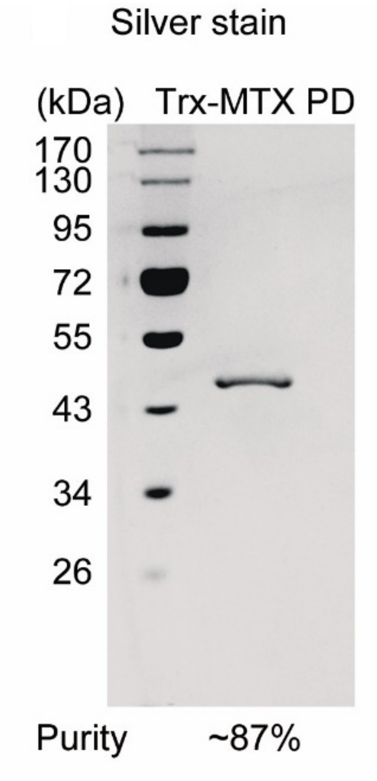

Figure S4

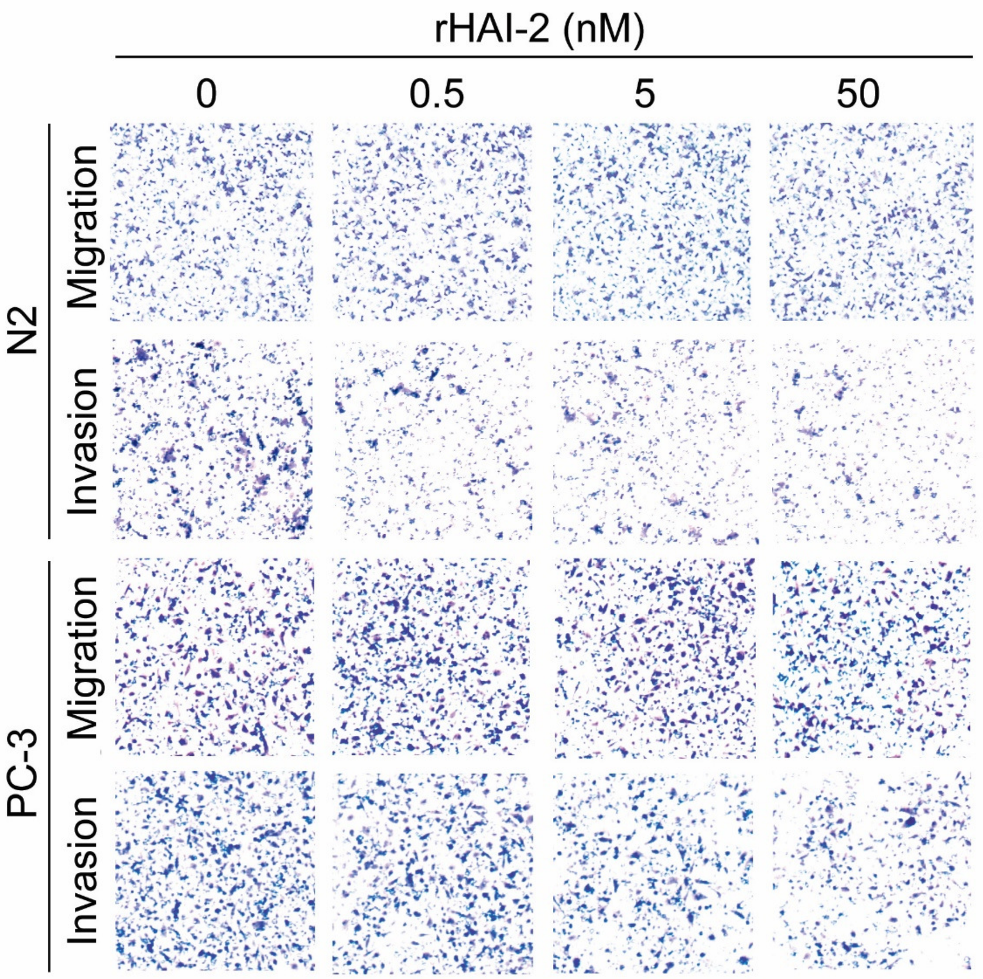

rHAI-2 (nM)
0
0.5
5
50
N2
Migration
Invasion
PC-3
Migration
Invasion

Figure S5

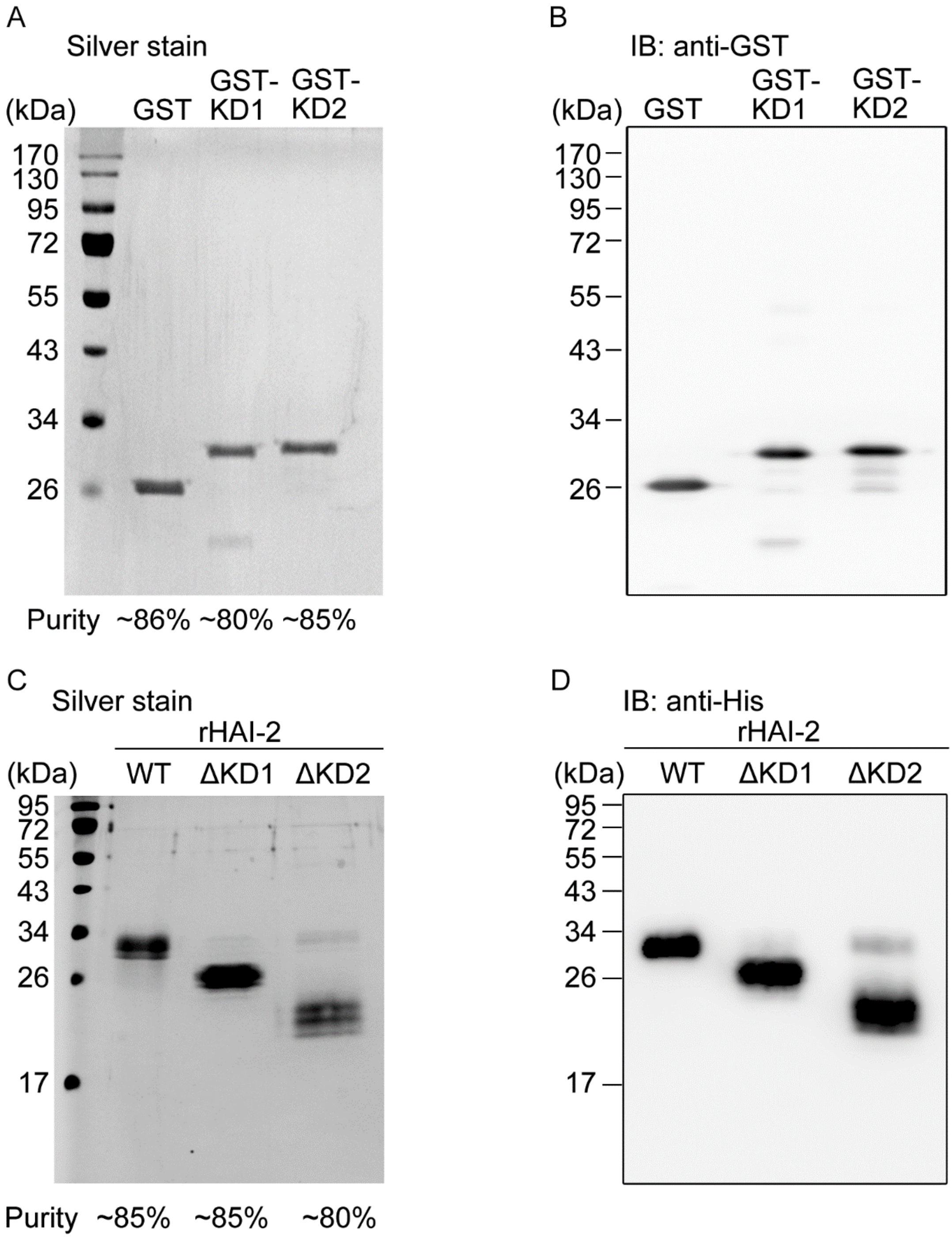
A
Silver stain
(kDa)
GST
GST-KD1
GST-KD2
170
130
95
72
55
43
34
26
Purity ~86% ~80% ~85%
B
IB: anti-GST
(kDa)
GST
GST-KD1
GST-KD2
170
130
95
72
55
43
34
26
C
Silver stain
rHAI-2
(kDa)
WT
ΔKD1
ΔKD2
95
72
55
43
34
26
17
Purity ~85% ~85% ~80%
D
IB: anti-His
rHAI-2
(kDa)
WT
ΔKD1
ΔKD2
95
72
55
43
34
26
17

Figure S6

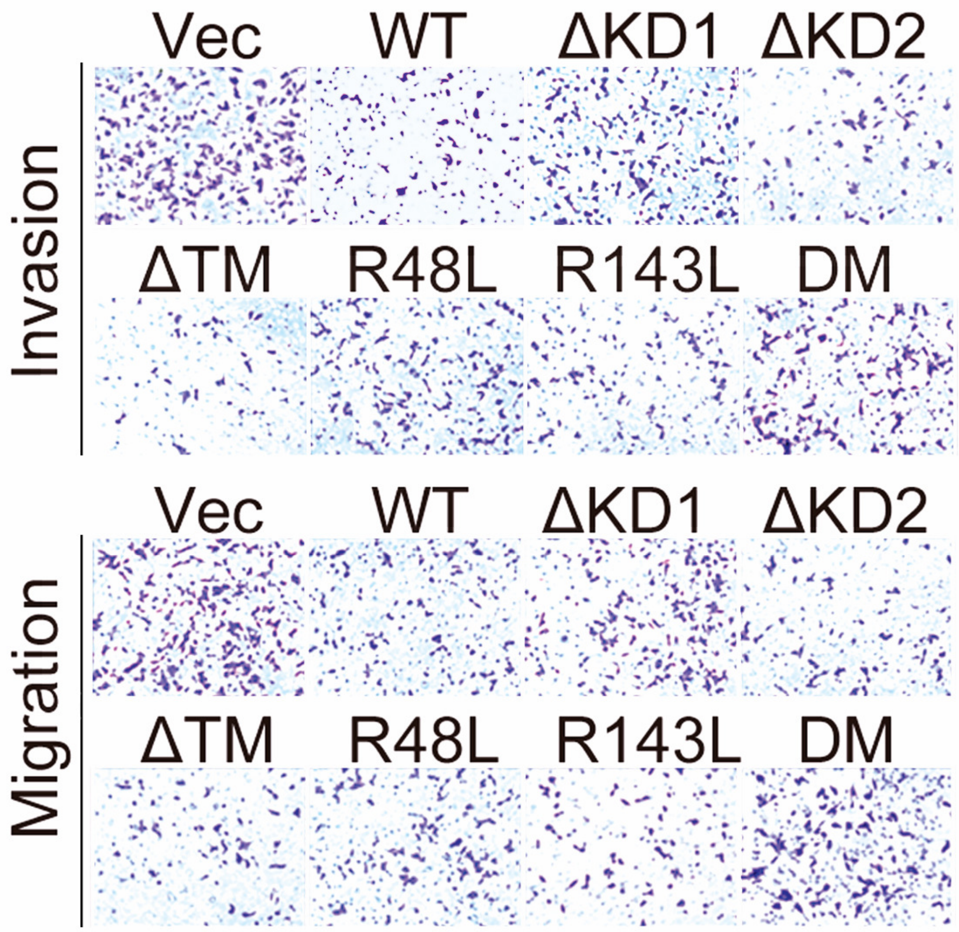

Vec
WT
ΔKD1
ΔKD2
Invasion
ΔTM
R48L
R143L
DM
Vec
WT
ΔKD1
ΔKD2
Migration
ΔTM
R48L
R143L
DM

Figure S7

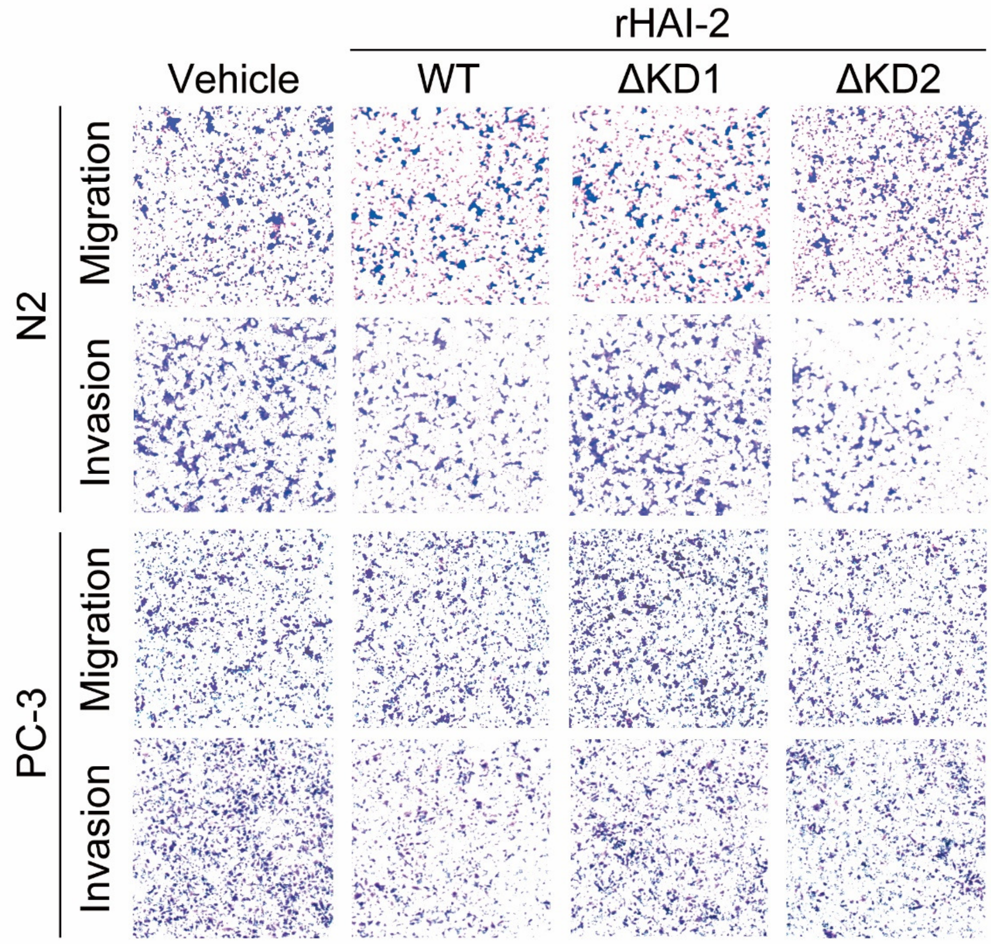
rHAI-2
Vehicle
WT
ΔKD1
ΔKD2
N2
Migration
Invasion
PC-3
Migration
Invasion

Figure S8

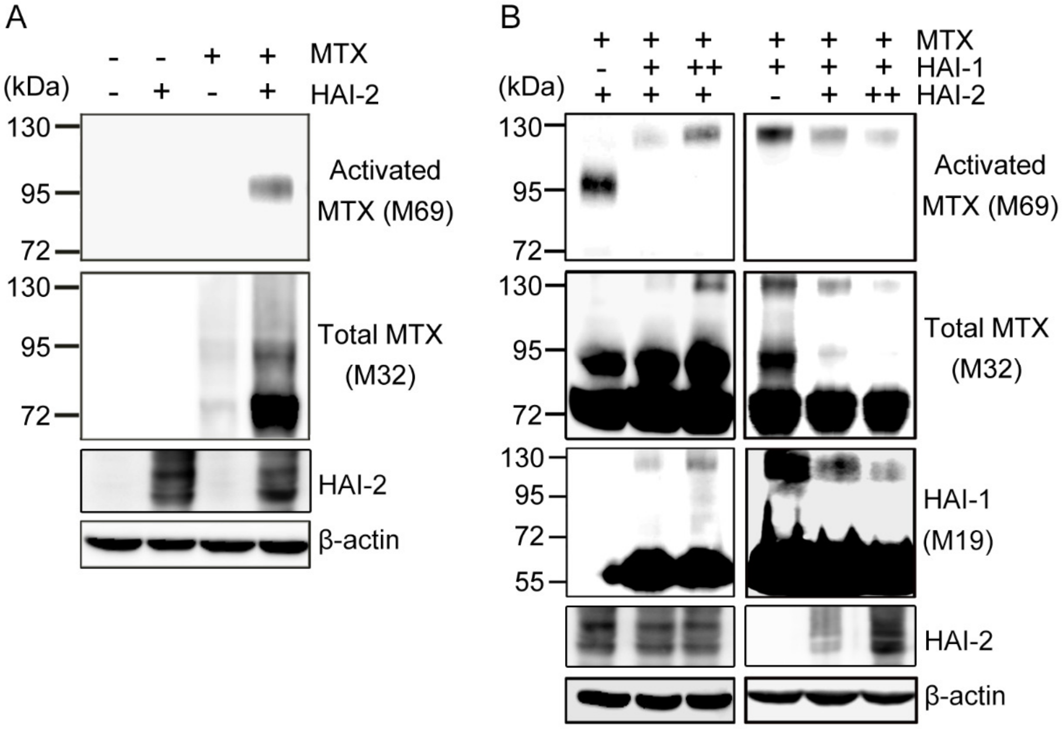

A
MTX
HAI-2
(kDa)
130
95
72
Activated MTX (M69)
Total MTX (M32)
HAI-2
β-actin
B
MTX
HAI-1
HAI-2
55
HAI-1 (M19)

Figure S9

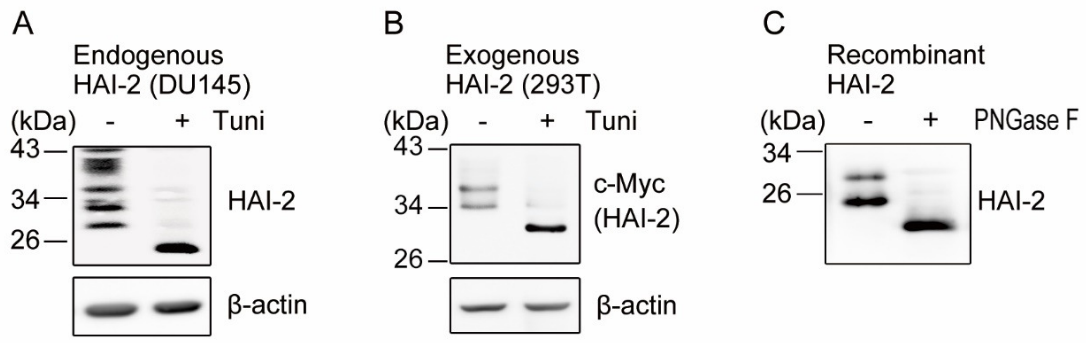
A
Endogenous
HAI-2 (DU145)
(kDa)
-
+
Tuni
43
34
26
HAI-2
β-actin
B
Exogenous
HAI-2 (293T)
(kDa)
-
+
Tuni
43
34
26
c-Myc
(HAI-2)
β-actin
C
Recombinant
HAI-2
(kDa)
-
+
PNGase F
34
26
HAI-2

Figure S10

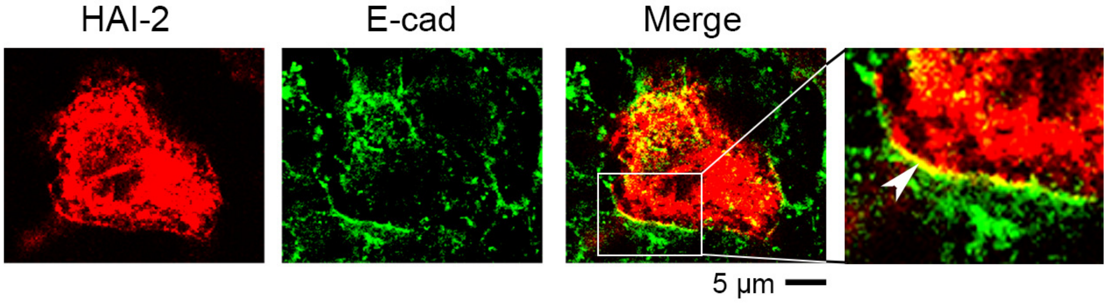

Figure S11

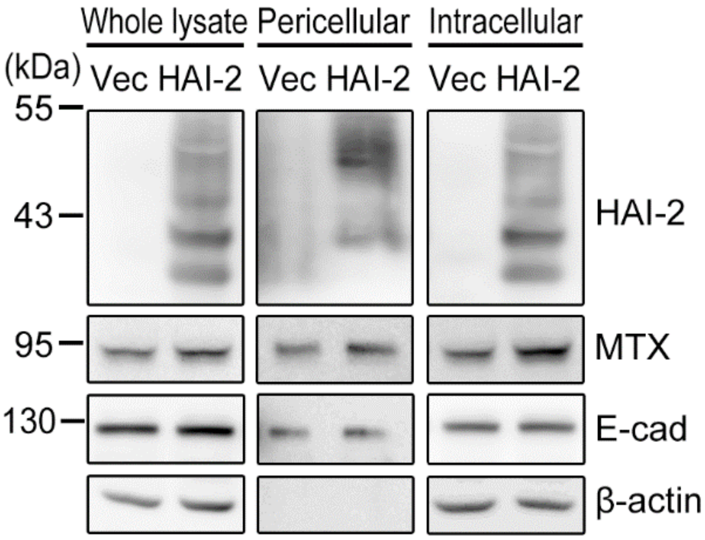
Whole lysate
Pericellular
Intracellular
(kDa)
Vec HAI-2
Vec HAI-2
Vec HAI-2
55
43
95
130
HAI-2
MTX
E-cad
β-actin

Figure S12

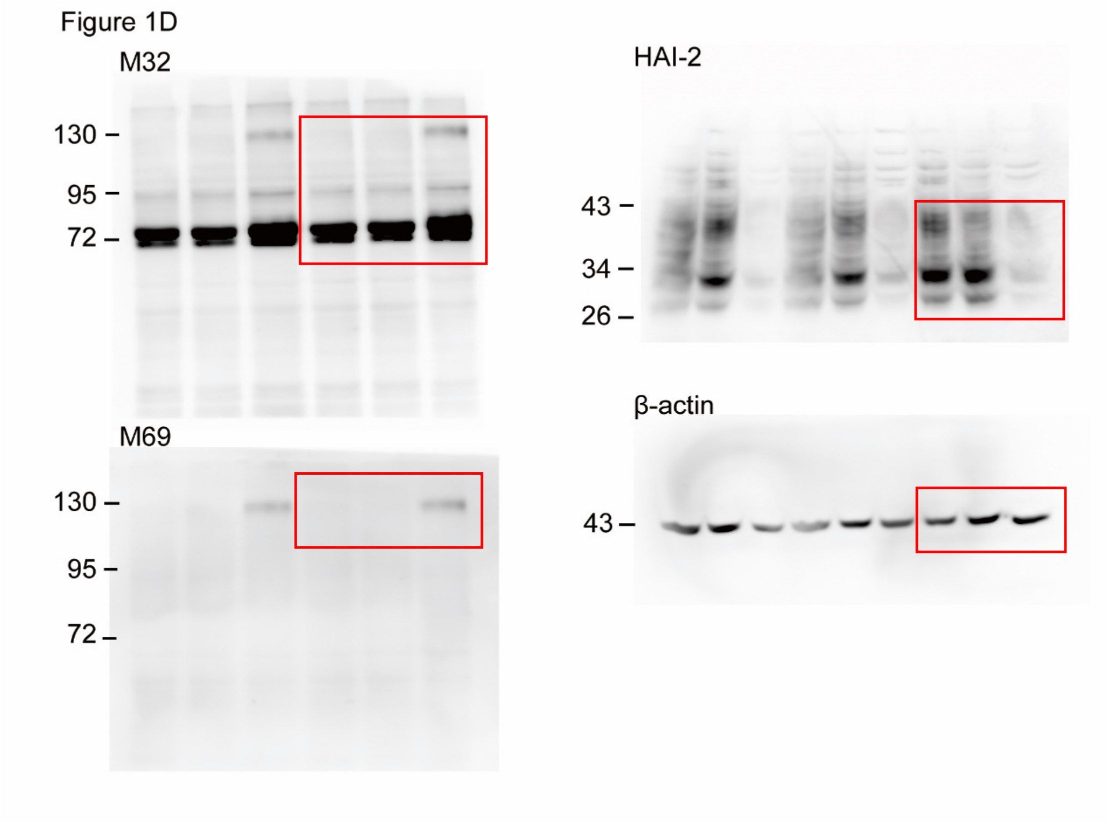

Figure S13

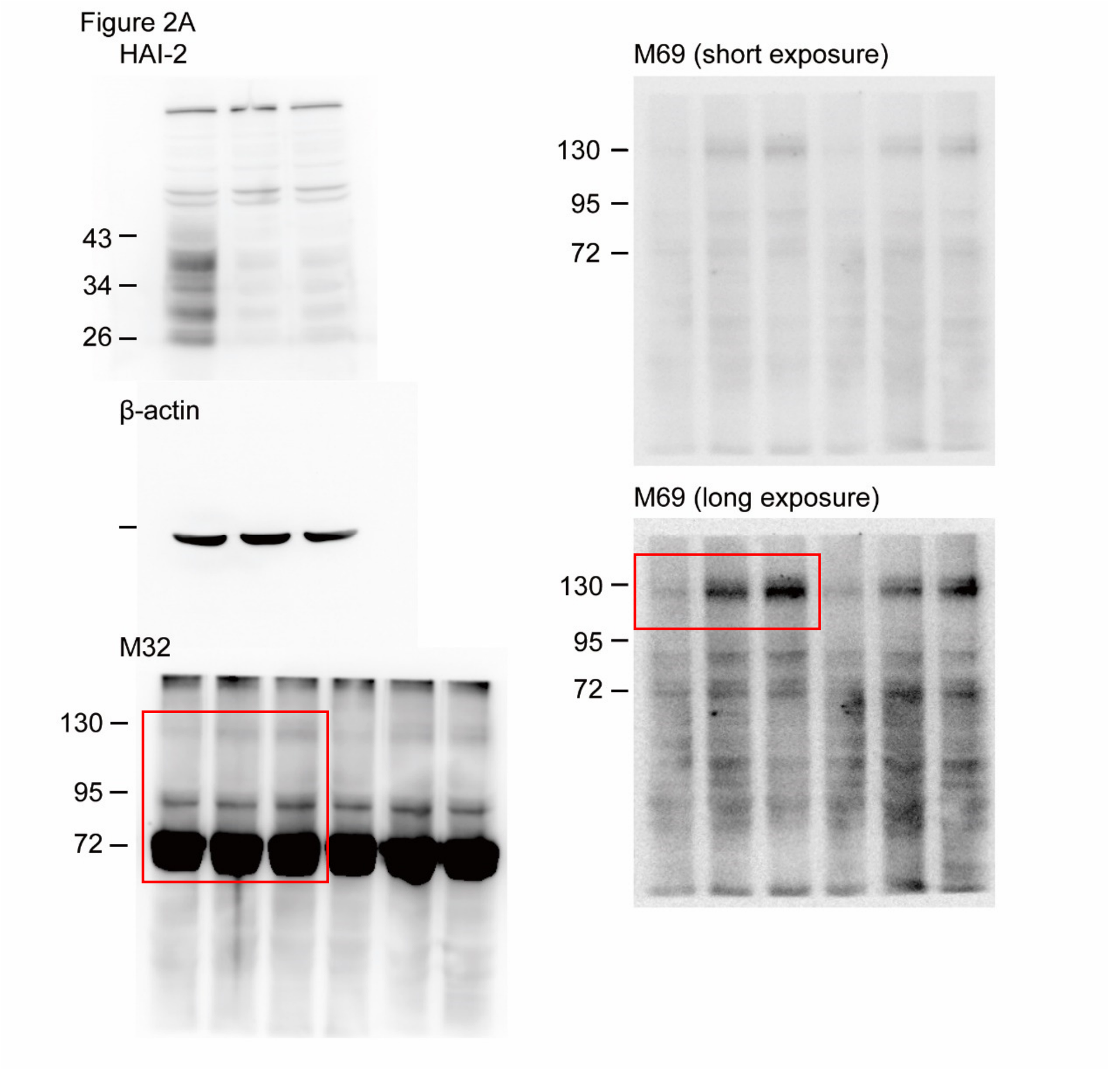

Figure 2A
HAI-2
43
34
26
β-actin
M32
130
95
72
M69 (short exposure)
130
95
72
M69 (long exposure)
130
95
72

Figure S14

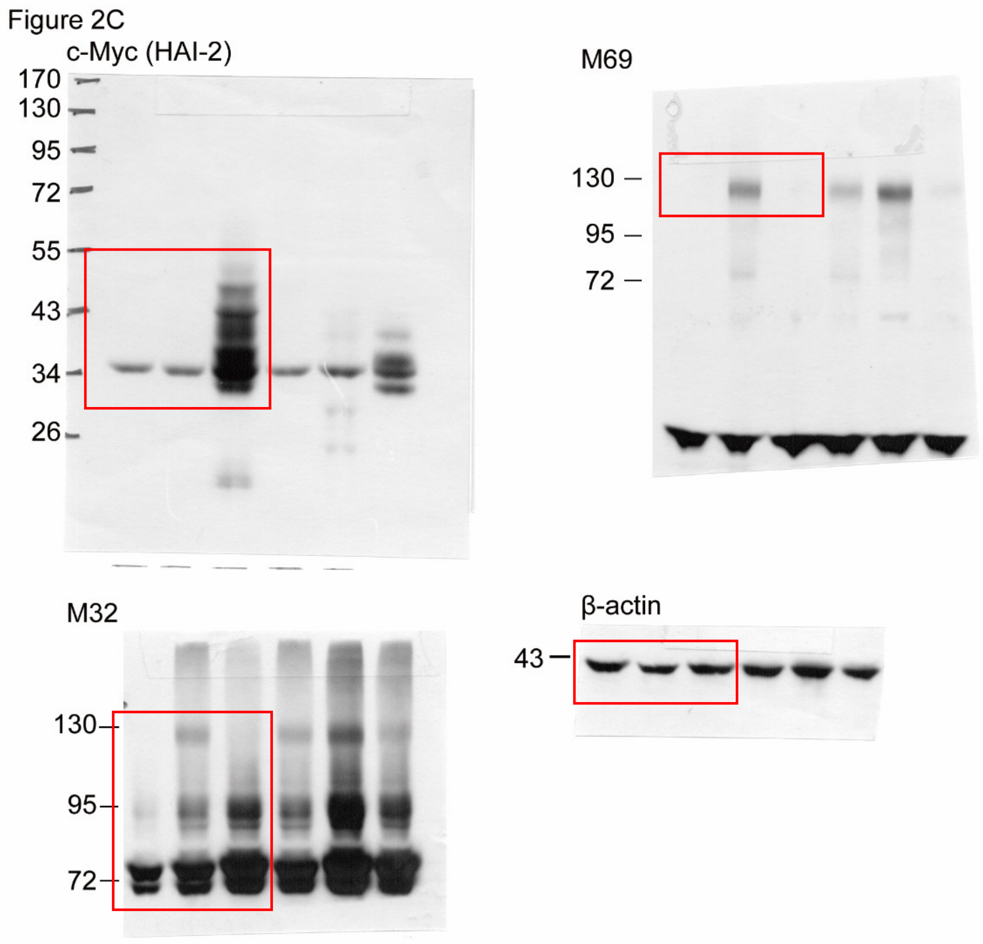

Figure 2C
c-Myc (HAI-2)
170
130
95
72
55
43
34
26
M69
130
95
72
M32
130
95
72
β-actin
43

Figure S15

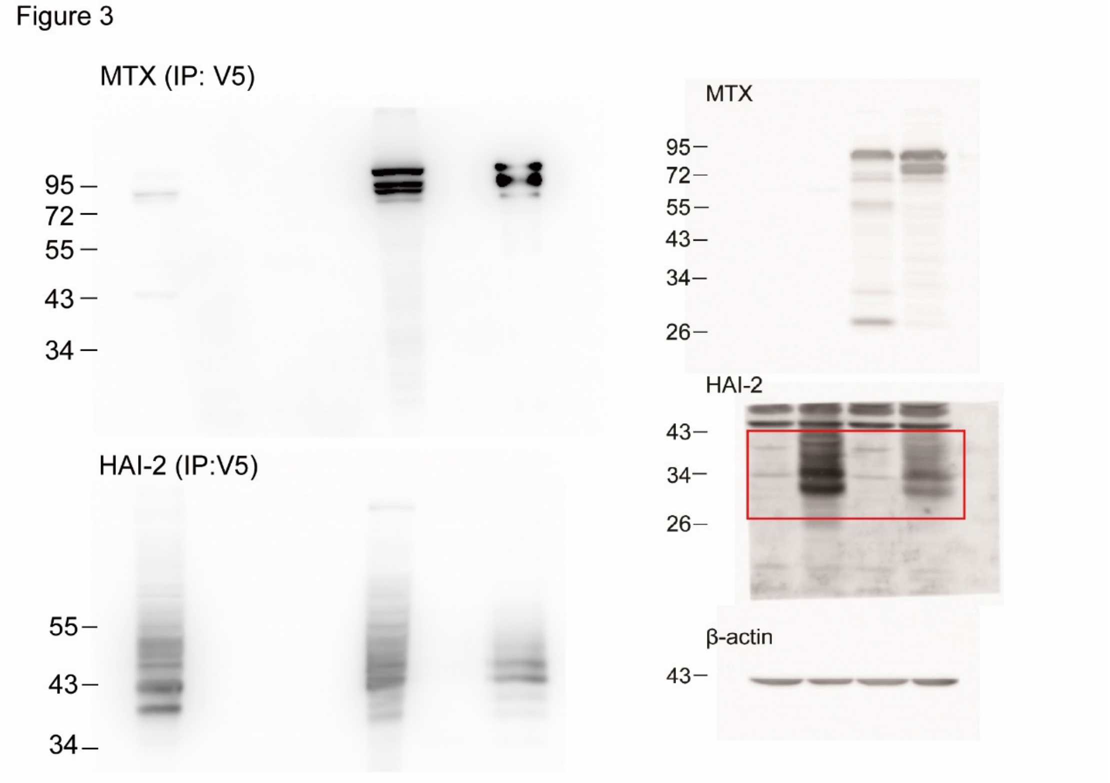
Figure 3
MTX (IP: V5)
95
72
55
43
34
HAI-2 (IP:V5)
55
43
34
MTX
95
72
55
43
34
26
HAI-2
43
34
26
β-actin
43

Figure S16

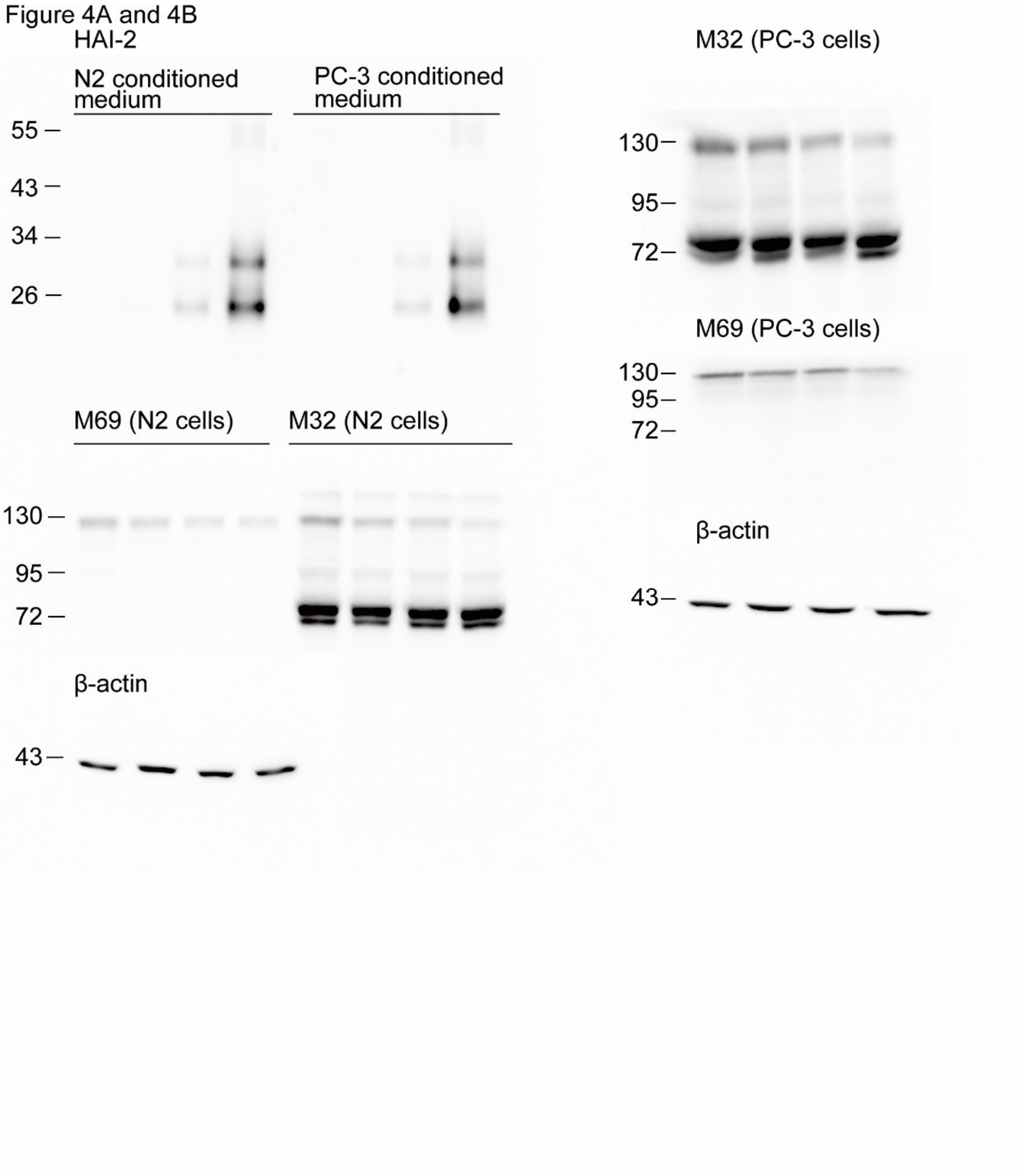
Figure 4A and 4B
HAI-2
N2 conditioned medium
PC-3 conditioned medium
55
43
34
26
M69 (N2 cells)
M32 (N2 cells)
130
95
72
β-actin
43
M32 (PC-3 cells)
130
95
72
M69 (PC-3 cells)
130
95
72
β-actin
43

Figure S17

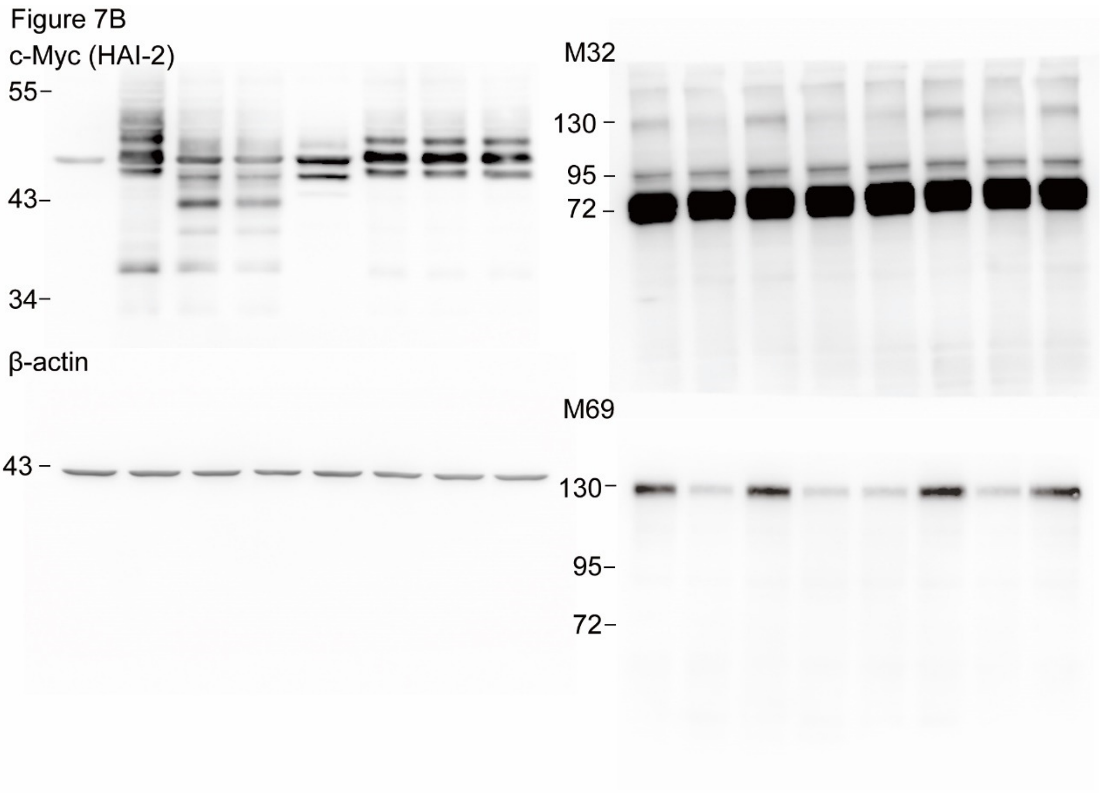
Figure 7B
c-Myc (HAI-2)
55–
43–
34–
β-actin
43 –
M32
130 –
95 –
72 –
M69
130–
95–
72–

Figure S18

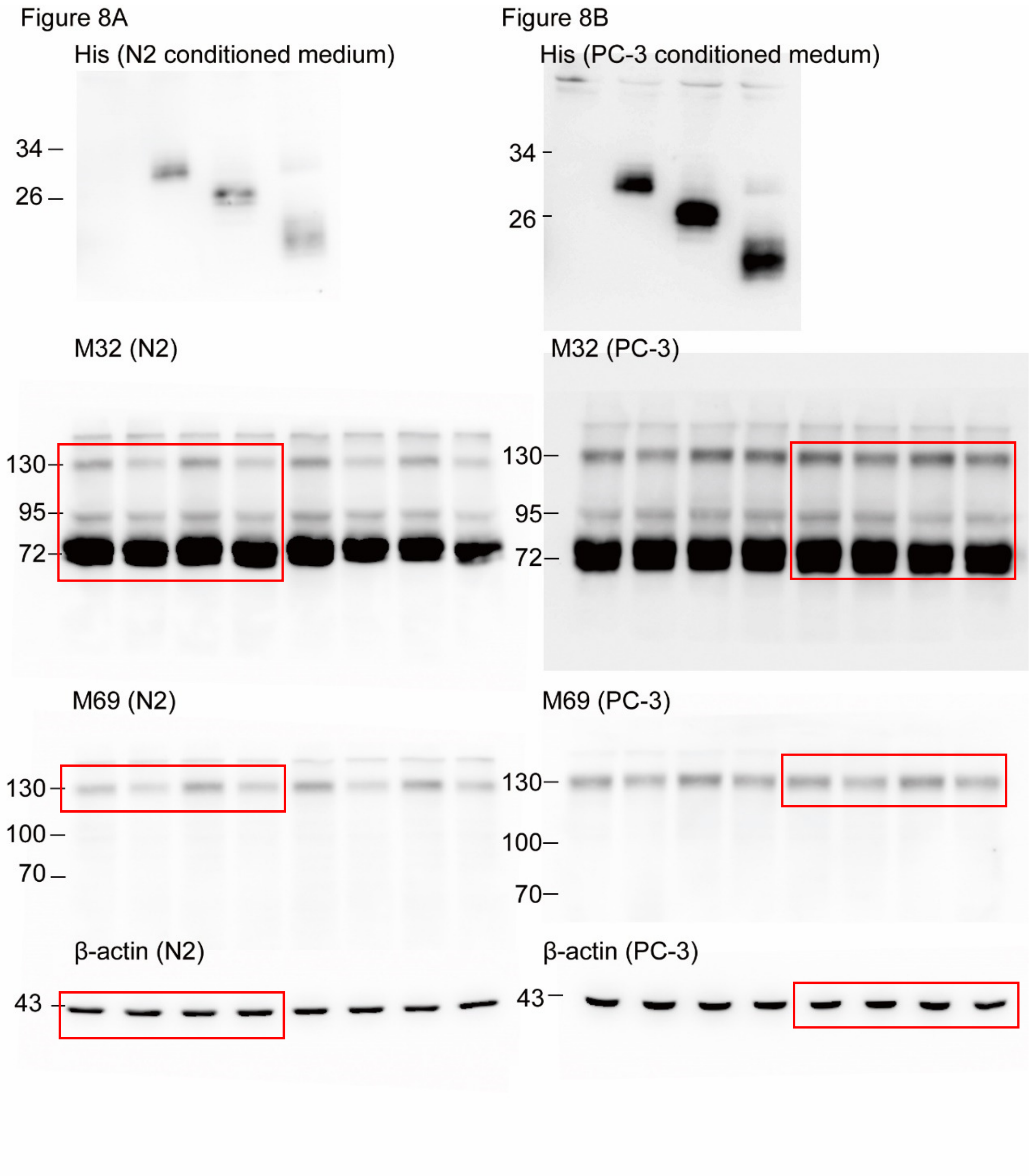

Figure 8A
His (N2 conditioned medium)
34
26
Figure 8B
His (PC-3 conditioned medum)
34
26
M32 (N2)
130
95
72
M32 (PC-3)
130
95
72
M69 (N2)
130
100
70
M69 (PC-3)
130
100
70
β-actin (N2)
43
β-actin (PC-3)
43

Table S1

Mutants	Sequence (5’->3’)
ΔKD1	Forward:<br>GAACGCAGCATCCACGACTTCACTGTCACAGAGAATGCCACG<br>Reverse:<br>CGTGGCATTCTCTGTGACAGTGAAGTCGTGGATGCTGCGTTC
ΔKD2	Forward:<br>AGCGATATGTTCAACTATGAACGCCAGCAGGAGAATCCTCCCCTG<br>Reverse:<br>CAGGGGAGGATTCTCCTGCTGGCGTTCATAGTTGAACATATCGCT
ΔTM	Forward:<br>CTGCCCCTTGGCTCAAAGGTGCGGGTGGCACGGAGGAACCAG<br>Reverse:<br>CTGGTTCCTCCGTGCAACCCGCACCTTTGAGCCAAGGGGCAG
R48L	Forward:<br>GTGGGCAGATGCCTGGCCTCCATGCC<br>Reverse:<br>GGCATGGAGGCCAGGCATCTGCCCAC
R143L	Forward:<br>CACTGGGCCTTGCCTTGCATCCTTCCC<br>Reverse:<br>GGGAAGGATGCAAGGCAAGGCCCAGTG
